# Feasibility and Process Evaluation of a Need-Supportive Physical Activity Program in Aged Care Workers: The Activity for Well-Being Project

**DOI:** 10.3389/fpsyg.2020.518413

**Published:** 2020-09-30

**Authors:** Merilyn Lock, Dannielle Post, James Dollman, Gaynor Parfitt

**Affiliations:** UniSA Allied Health and Human Performance, University of South Australia, Adelaide, SA, Australia

**Keywords:** physical activity, need support, self-determination theory, affective valence, rating of perceived exertion, process evaluation, aged care workers

## Abstract

The need to undertake pilot testing and evaluation of novel health promotion programs has become increasingly apparent for the purpose of understanding the true effects of complex interventions and for testing and refining behavioral theories that these interventions are informed by. A mixed-methods process evaluation and feasibility study was undertaken for a need-supportive physical activity program that was piloted in a single-group pre–post study. The piloted program was designed to support participant needs of autonomy, competence, and relatedness through evidence-based and theory-informed behavior change strategies including a motivational interviewing style appointment, education on self-management tools (i.e., pedometers, goal setting, action and coping planning, a customized website for goal setting and self-monitoring), and self-determined methods of regulating physical activity intensity [affect, rating of perceived exertion (RPE), and self-pacing]. The program aimed to positively impact physical activity behavior, psychological well-being, and associated motivational processes. Reach, adoption, fidelity, context, change and performance objectives, and feasibility of the program were evaluated using information from survey respondents from the target population (*n* = 118) and implementing staff (*n* = 6); questionnaires from pilot study participants (*n* = 21); and individual semi-structured interviews with a combination of pilot study participants, non-participants, and implementing staff (*n* = 19). Process evaluation of the Activity for Well-Being program found that the reach of the program was moderate but adoption was low. The use of self-management tools and self-determined methods of regulating physical activity intensity appeared to be feasible. The website had mixed responses and low engagement. The element of having a support person elicited a strong positive response in the program participant interviews. Involving local implementing staff more directly into the delivery of the intervention could have potentially improved reach, adoption, and feasibility of the program.

## Introduction

A large body of research has been devoted to investigating the effects of physical activity interventions in the workplace over the past four decades, many of these being complex behavior change interventions (Dugdill et al., 2008; Abraham and Graham-Rowe, 2009; Malik et al., 2013). Complex interventions have been described as interventions that contain several interacting components (Craig et al., 2008; Moore et al., 2015). The relationships between the mechanisms of behavior change, the implementation of the intervention, and the context within which it is being implemented are critically important to the impact and outcomes of the intervention (Moore et al., 2015). Many factors may influence outcomes that would make it difficult to determine what elements are at play when testing the effectiveness of an intervention in a real-world context (Shepperd et al., 2009; O’Brien et al., 2015). These factors may include the variability of the population or outcomes, the number of groups or organizational levels targeted by the intervention, and the degree of flexibility or tailoring of the intervention (Craig et al., 2008); traditionally, these factors have not been accounted for in program evaluation. Quested et al. (2017) noted that intervention implementation was not a significant focus for the majority of identified physical activity interventions informed by motivational theory, while Wierenga et al. (2013) reported only 7.2% of studies investigating the effects of workplace health promotion programs also published a process evaluation. Similarly, in their systematic review of reviews, Greaves et al. (2011) noted that of 30 included literature reviews, none accounted for fidelity. These reviews emphasize the need for better evaluation of the implementation process, fidelity, and other contextual factors. The Medical Research Council (*London, United Kingdom*), as an organization that provides funding for preventative health research, has developed and refined guidance to account for these elements through process evaluation (Moore et al., 2015). The use of process evaluations is being increasingly recognized as important for ensuring scientific rigor (Glasgow et al., 1999; Issel and Wells, 2017; Glasgow et al., 2019) and also for clearly testing and refining behavioral theory (Brug et al., 2005; Dombrowski et al., 2007).

In addition to evaluation, Hoddinott (2015) outlined the importance of having systematic and transparent development of interventions that can then be tested in pilot studies and then refined before full efficacy testing in the form of a randomized controlled trial. Many behavior change interventions that have been tested in full randomized controlled trials have reported a basis in behavioral theory; however, the true extent to which theory was actually used to inform these interventions is often unknown. Past reviews of behavioral interventions have identified that, although studies may claim to be informed by behavioral theory, often the application of the theory within the development of the interventions is not methodical or thorough (Painter et al., 2008; Prestwich et al., 2014). This knowledge has led to an increased emphasis on the systematic development and evaluation of behavioral interventions, including the methodical application of behavioral theory within the development process.

Intervention mapping provides a systematic, six-step process for ensuring better quality and more explicit integration of health psychology theory into intervention design (Bartholomew et al., 1998; Kok et al., 2004; Bartholomew et al., 2011). The steps for the development and evaluation of behavioral interventions include (i) the implementation of a needs assessment; (ii) the preparation of matrices of change and performance objectives; (iii) the selection of theory-informed intervention methods and practical applications; (iv) the production of program components and materials; v) the planning of program adoption, implementation, and sustainability; and (vi) the planning for evaluation (Bartholomew et al., 2011). It is thought that more explicit use of theory within the development of the behavioral interventions ensures a higher quality of testing theoretical constructs and may also have the potential to improve the efficacy of the targeted interventions (Taylor et al., 2012). As such, frameworks like intervention mapping may prove valuable for ensuring systematic and transparent development of theory-informed interventions and subsequently the progression of the field of behavior change theory.

The Activity for Well-Being program was developed taking guidance from the intervention mapping framework, and the behavior change strategies used were based on self-determination theory (Deci and Ryan, 2000; Ryan and Deci, 2000). The application of self-determination theory within behavior change interventions tends to focus on psychological need support (i.e., Levy and Cardinal, 2004; Edmunds et al., 2006; Friederichs et al., 2016). As a theory, it has shown promise for effective physical activity behavior change, with a growing number of studies demonstrating positive relationships between need satisfaction and physical activity behavior, through the use of a need-supportive approach (Fortier et al., 2007; Weman-Josefsson et al., 2015).

Need support has also been seen to be positively related to psychological well-being (Edmunds et al., 2008; Ng et al., 2013) and exercise-related affect (Edmunds et al., 2008), another important predictor of physical activity behavior. Affect measured during exercise has been shown to predict future physical activity behavior (Williams et al., 2008; Rhodes and Kates, 2015), but only a limited number of studies have applied affect as a method of regulating activity intensity (Rose and Parfitt, 2008; Hamlyn-Williams et al., 2015). Strong arguments can be posed for the use of approaches such as self-pacing or preferred intensity (Parfitt et al., 2006; Williams, 2008; Vazou-Ekkekakis and Ekkekakis, 2009); however, little is known about the true impact of these approaches on motivation and behavior in a real-world context. In theory, strategies such as self-pacing or the use of affect or rating of perceived exertion (RPE) may provide a method of regulating physical activity intensity that align well with self-determination theory and are inherently supportive of the participant’s autonomy (Williams, 2008; Rhodes and Kates, 2015).

Acknowledging all of these factors, the Activity for Well-Being project extended the work of previous need-supportive interventions by ensuring the explicit application of theory within the development of the intervention, including an emphasis on the use of more self-determined methods of regulating activity intensity and evaluating fidelity and feasibility of the project. This paper presents the findings of the mixed-methods process and feasibility evaluation of the project.

The objectives of the current study were as follows:

(1) To evaluate the reach, adoption, fidelity, and context related to the implementation of the Activity for Well-Being program in the form of a pilot intervention trial.
(2) To investigate the feasibility and limitations of the program for the target population of frontline aged care workers and to provide recommendations and guidance regarding the future directions for the program.

## Materials and Methods

**FIGURE 1:**
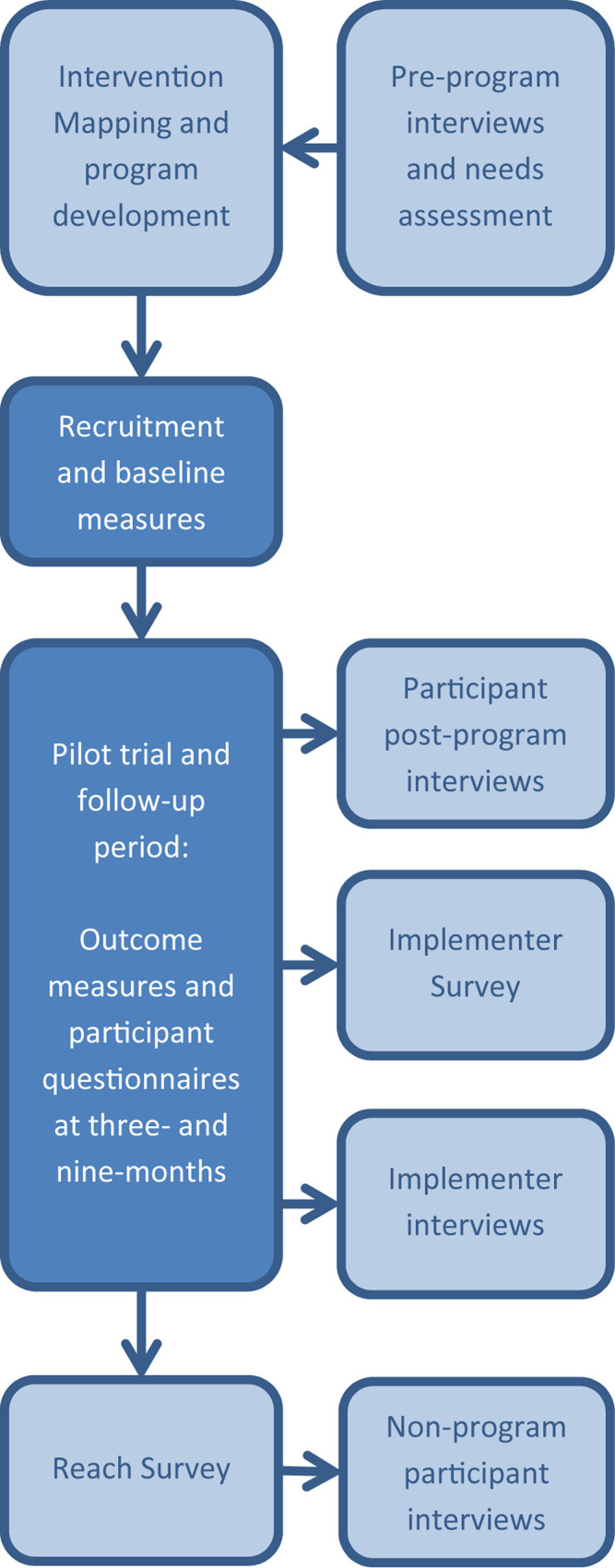
Schematic of the project implementation and data collection for process evaluation.

The Activity for Well-Being program was run as a single-group pre–post pilot trial with follow-up measures at 9 months. Methods of evaluation drew from multiple well-established guidelines and frameworks (Glasgow et al., 1999; Bartholomew et al., 2011; Moore et al., 2015; Issel and Wells, 2017). Data collection for the process evaluation occurred concurrently with the pilot testing of the program and continued until after the 9-month follow-up period was completed. The process evaluation and feasibility study for the Activity for Well-Being program used a mixed-methods approach including quantitative questionnaire and survey data, and qualitative semi-structured individual interviews. Reach and Implementer Surveys were sent out to all frontline employees and implementing staff, respectively, after the completion of the Activity for Well-Being program. Reach Surveys were distributed to community-based workers via an online survey and via a paper survey for residential employees who had limited access to emails at work. A detailed description of the methods for the development and evaluation of the program, including the intervention mapping approach and findings of the needs assessment, has been outlined previously (Lock et al., 2018). Figure 1 provides a schematic of the structure of the project and process evaluation. Ethical approval for this study was obtained from the University of South Australia Human Ethics Research Committee, and the trial was preregistered with the Australian and New Zealand Clinical Trials Registry (registration number: ACTRN12617001395325).

### Participants and Recruitment

All participants in the pilot program, process evaluation, and feasibly study were employees of a large not-for-profit, aged care organization in the metropolitan and surrounding regional areas of Adelaide, South Australia. The process evaluation and feasibility study outlined here included survey and interview data from *program participants*, *non-program participants*, and *implementing staff*.

*Program participants* included community- and residential-based aged care workers who chose to participate in the pilot study of the Activity for Well-Being program during the period of November 2017–April 2019. Frontline aged care workers were identified as the target population during the initial needs assessment and consultation with the funding organization. Program participants were recruited from all community-based areas and three residential sites using a combination of emails, posters, flyers, staff newsletter entries, and face-to-face introductions at staff meetings (undertaken by the primary researcher or local implementing staff). Community-based employees were primarily recruited through emails and team meetings, since they had limited time in an office or physical space and received most communications on work phones or devices. Residential employees were primarily recruited through the use of posters/flyers or newsletter entries, as well as staff meetings, since these employees worked in a regular location but had limited access to work emails. All program participants provided informed, written consent for the program and the interviews at baseline, at which point they indicated whether they would like to be contacted regarding an interview. Program participants were invited to participate in an interview during the period between the 3- and 9-months measures but were under no obligation to accept the invitation.

*Non-program participants* consisted of members of the target population (frontline aged care workers) who did not participate in the pilot study of the program. Non-program participants were recruited via an online survey (the Reach Survey) that was sent out after the conclusion of the pilot study. Consent for this survey was implied by the respondent completion of the survey, while all participants gave recorded verbal informed consent at the start of the interview. All survey respondents had access to an online version of the participant information for interviews and were given the chance to opt-in to an interview during the Reach Survey process. Interviews were offered to survey respondents who had “opted-in” until data saturation was reached. Purposive sampling was used to include participants with a range of responses to survey questions in interviews.

*Implementing staff* consisted of any employee involved with the implementation or promotion of the program within the local site or location. These staff may have included managers or assistant managers, team leaders, staff involved with healthy aging and health promotion (i.e., gym staff or therapy assistants), administration staff, or others. All implementing staff members were emailed directly with a copy of the participant information and an invitation to participate in the interviews.

For all invitations to participate in interviews (to program participants, non-program participants, and implementing staff), the researchers made it clear that invited staff were under no obligation to accept the invitation. For all interviews, points of consent were read, and clarified where necessary, and written (for face-to-face interviews) or verbal (for telephone interviews) consent was obtained prior to initiating any interviews. All Reach Survey respondents went into a draw to win a $50 AUD gift card (with one in 10 chance of winning), and all interview participants received a gift card honorarium of $30 AUD in appreciation of their participation.

### Overview

The Activity for Well-Being program was developed using a participatory approach guided by the intervention mapping framework (Bartholomew et al., 1998, 2011) and was based on self-determination theory (Deci and Ryan, 2000; Ryan and Deci, 2000). As a part of the development process, a needs assessment was undertaken that included pre-program interviews with members of the target population (frontline aged care workers, *n* = 10, all community based) (Lock et al., 2018). In brief, the program was based on self-determination theory and used a number of evidence-based strategies including: a motivational interviewing style initial appointment delivered by an accredited exercise physiologist (AEP) with masters-level training in motivational interviewing (a common strategy used within AEP practice in Australia). Program participants also received education on goal setting, action and coping planning, self-monitoring via a pedometer or own wearable device, and education on the use of self-determined methods of activity intensity regulation (affect, RPE, and self-pacing). To facilitate the use of these methods of regulating physical activity intensity, participants were given a business size card with Hardy and Rajeski’s Feeling Scale (Hardy and Rejeski, 1989) and Borg’s RPE scale (Borg, 1982) to keep, were given the opportunity to use the scales during the initial appointment, and were provided instructions as to how to use the scales and self-pacing for regulating physical activity intensity.

Participants were also provided with ongoing physical activity support by the AEP, including need-supportive follow-ups, exercise prescription for home- or gym-based programs, face-to-face assistance with initiating exercise programs, and sign posting to community activities depending on the goals and preferences of the participant. Support was actively offered for the 3 months of the active program period, initiated via two need-supportive follow-up communications (at around weeks 2 and 8, unless initiated by the participant prior). For the follow-up period of 6 months, support was no longer actively offered by the AEP, but participants were able to contact the AEP for support until the end of the follow-up period (the 9-month time point). This approach was taken in order to promote autonomy in activity management within the program participants without withdrawing all need support. Participants also received access to a website, previously piloted (Frensham et al., 2014) and modified for the target population, and monthly informational newsletters for the entire 9-month period. The website had a function for tracking step counts, setting tiered goals (three different weekly step goals based on whether the participant was feeling “good,” “okay,” or “bad”), and links to health information and local community activities.

The process evaluation of the program included assessments of reach, adoption, fidelity, and context. Outcome, performance, and change objectives that were created through the intervention mapping process were evaluated for program participants through a questionnaire at 3 months. The feasibility of the program was evaluated through qualitative and quantitative research methods and included elements of attrition, adherence, subjective outcomes, program components, and sustainability. The theoretical mechanisms of behavior change were evaluated as process measures, along with the outcomes, and are not within the scope of this paper.

### Process Evaluation

#### Reach and Adoption

After the completion of the program, the online Reach Survey was sent to all frontline workers from all sites and areas that were originally invited to participate. Reach was calculated as the percentage of the population aware of the program. Additional information gathered from the survey included the following: *would the Activity for Wellbeing program have been something you would have been interested in participating in, had you known about it*; *what do you feel would have been the best way to promote the program*; and *why did you choose to/not to participate in the program?* Adoption was calculated as the percentage of the population who chose to participate in the program. Demographic data for the program participant sample were compared with the whole population data and data for individual sites and areas, where available. An Implementer Survey that was sent to all implementing staff at the same time as the Reach Survey was distributed. This survey assessed the level of knowledge about the nature of the program by allowing the respondent to select different components of the program according to their understanding. Other questions included in this survey were as follows: *do you feel that the program had value for improving the health and wellbeing of your employees*; *during the course of the program, did you feel that the program was something that you promoted to your employees or encouraged participation in*; *did you notice any impact of the program*; *what do you feel may have been the greatest barrier(s) to participation*; *what do you feel were the main incentives to participate*; *do you feel that it would be valuable to maintain the use of a program such as this in the future*; and *do you feel that it would be valuable to use a different type of program in the future?* Information gathered from this survey was used to compare differences between community and residential locations.

#### Fidelity

Fidelity of the implementation of the initial appointments was evaluated through consented recordings of four randomly selected initial appointments (16% of total). These recordings were evaluated by a member of the research team who was highly proficient in motivational interviewing but not directly involved in the implementation of the program or delivery of the interviews. The interviews were assessed using a pre-prepared fidelity checklist that scored 15 components as achieved, not achieved, or not applicable. These components included education around six program components (goal setting, action and coping planning, and the use of the pedometer, website, and self-determined forms of regulating activity intensity); four elements relating to the collaborative development of the program (the use of participant preferred activities, whether the participant perceived the activity plan as achievable as measured on a confidence scale of 1–10, whether the plan was adapted and confidence re-measured when necessary, and if activities were not imparted on the participant without participant engagement or consent); and five fundamentals of the initial appointments (use of empathy, supporting and developing discrepancy, rolling with resistance, supporting self-efficacy, and supporting autonomy), emphasizing autonomy support and principles of motivational interviewing (Miller, 1983; Rollnick and Allison, 2004). The support of autonomy, as a fundamental element of a need-supportive approach, was additionally measured with the Health Care Climate Questionnaire (HCCQ) (Williams et al., 1996).

#### Intervention Mapping Evaluation

Evaluation of the intervention mapping components included outcome objectives (behavioral, well-being, and interpersonal environmental), performance objectives, and change objectives. The results of the behavioral, well-being, and interpersonal environmental outcomes, along with additional process and performance measures, will be reported elsewhere.

Specific performance objectives and change objectives were developed during the program development using guidance from the intervention mapping framework. Performance objectives were three “sub-behaviors” of the target behavior (physical activity participation) that were identified as important for this population during the pre-program needs assessment. These performance objectives were as follows: find time to undertake physical activity, find motivation to undertake physical activity, and identify opportunities to undertake physical activity. Twelve change objectives were developed as composites of these sub-behaviors and four targeted determinants of behavior (perceived autonomy, perceived competence and perceived relatedness in exercise, and positive exercise affect). The matrices of performance and change objectives can be seen in Supplementary Material 1.

Each of the performance and change objectives was assessed through a questionnaire that was completed at the 3-month data collection point. The achievement of performance objectives was assessed using questions that specifically related to the objectives, e.g., for the performance objective “find time to undertake physical activity,” the evaluating question was “do you generally feel you are better able to find time to be active?” Change objectives were assessed using a small cluster of questions developed within the domains of the targeted personal determinants (support for autonomy, competence, and relatedness). Participants rated each objective-related question on a five-point Likert scale, where 1 = not at all and 5 = very much so. Since “3” could be considered a “neutral” score between “1” and “5,” scores of “1” or “2” could be considered negative changes, and scores of “4” or “5” could be considered positive changes. Mean scores for the whole participant sample were calculated for each of the single-item performance objectives and composite change objectives. Groupings of questions for the change objectives were assessed for internal consistency using Cronbach’s alpha (α) in SPSS (version 25.0; IBM Corp., Armonk, NY). In the cases where the questions were deemed to contain elements of more than one domain, groupings were refined based on best fit. Questions that demonstrated a poor fit within all groups were excluded from the final groupings.

#### Feasibility, Context, and Qualitative Methods

Qualitative research methods were used to gather information to support the quantitative data outlined for each of the different components of the process evaluation. In addition to this, qualitative methods were used to assess the overall feasibility of the program for the target population as well as the context in which the program was implemented. Individual telephone or face-to-face interviews were undertaken with program participants, non-program participants, and implementing staff. Interviews used a semi-structured, emergent-systematic approach. Interviews with program participants investigated elements of the program that the participant felt did or did not work, as well as the perceived impact of the program from the perspective of the individual program participant. The following open-ended facilitating questions were used (and adapted where necessary) to initiate discussion and address the objectives of the study: *What aspects of the program were helpful for increasing/maintaining physical activity levels? What aspects of the program were not helpful? Do you think you have you maintained physical activity levels since the end of the three-month program? What has helped you/stopped you from doing this?*

Interviews with implementing staff explored feasibility and perceived impact of the program from their perspective. The primary facilitating questions for the implementing staff were as follows: *What aspects of the recruitment for the program were effective? What aspects of the recruitment were not effective? How could the recruitment for the program be improved? What aspects of the implementation of the program were effective? What aspects of the implementation were not effective? How could the implementation of the program be improved? Do you have any other feedback about the program?*

Interviews with non-participants focused on the reasons for choosing not to participate in the program or other well-being programs offered by the organization. The primary facilitating question for non-participant interviews was as follows: *what are your reasons for choosing not to participate in the program?* Information on the level of awareness of the program, the recruitment methods, and the work context within which the program was being implemented was drawn from emergent conversation that developed across all (program participant, implementer, and non-participant) interviews. All interviews were undertaken during the period of April 2018–May 2019.

### Data Processing and Analysis

For the purposes of the process evaluation, the data from all surveys and questionnaires were analyzed using descriptive statistics. Audio recordings of interviews were transcribed and imported into NVivo qualitative analysis software, version 12.0 (*QSR International*). All interviews were analyzed using a structured thematic approach (Braun and Clarke, 2013). Interview transcripts were coded thoroughly by two members of the research team. The coding was undertaken by allocating sections of speech from within the transcripts to nodes developed within the NVivo software. Most sections of speech were coded to multiple nodes that were then refined as themes and sub-themes. During the refinement process, some similar codes were merged and some codes were separated. Overarching themes were developed for sorting codes into factors that influenced program adherence, different elements of the process evaluation (reach, adoption, sustainability, and context), and information relating to specific program components (i.e., self-management tools or activity intensity regulation). Within the overarching themes of reach, adoption, and adherence, codes were separated into factors that had a positive influence (facilitators) and those that had a negative influence (barriers). Codes within the other overarching themes were sorted into themes and sub-themes based upon the content. Information pertaining to adherence, program components, subjective outcomes of the program, and sustainability was used to inform the feasibility component of the evaluation.

## Results

### Process Evaluation

#### Participants and Recruitment

A total of 118 employees responded to the Reach Survey including 99 community-based workers and 19 residential workers. The Implementer Survey was completed by six of the 12 implementing staff directly involved with the program, including three residential-based workers, two community-based workers, and one staff member involved with implementation across both residential and community settings. Interview participants included 10 pilot program participants (nine community based and one residential based), six non-program participants (four community based and two residential based), and three implementing staff (one community based and two residential based).

The pilot trial of the Activity for Well-being Program was finalized with 25 participants. One participant withdrew from the program at 3 months, and two participants withdrew at 9 months. No participants who withdrew from the program gave consent to an interview; however, the reasons that were given for the withdrawal from the program included pregnancy (*n* = 1) and time constraints (*n* = 2). The results of the mixed-methods process evaluation and feasibility study are outlined here. Themes, sub-themes, and selected quotes from the interviews can be found in Supplementary Material 2.

#### Reach and Adoption

The primary findings of the Reach Survey and recruitment, including levels of awareness, preferred modes of promotion, and reasons for participating or not participating in the pilot program, can be seen in Table 1.

**TABLE 1:** Elements of reach and adoption informed by survey responses and recruitment.

	**Total**	**Community**	**Residential**
**Population**			
Employees invited to participate in the program and to complete the Reach Survey	493	287	206
**Reach**			
Responded to Reach Survey	118	99	19
(% of total population)	(23.94)	(34.49)	(9.22)
Primarily voluntary employee	4	2	2
Aware of program	70	57	13
(% of respondents)	(59.32)	(57.58)	(68.42)
Not aware of program, unsure, or no response	48	42	6
(% of respondents)	(40.68)	(42.42)	(31.58)
Would have been interested had they known about it	22	18	4
*Preferred mode of promotion*			
Email	26	23	3
Team meetings	25	22	3
Flyers/written material	17	12	5
Newsletters	8	7	1
Including more information	7	7	0
SMS	7	6	1
“Same as was done”	5	3	2
Compulsory training	4	4	0
Other	21	15	6
**Adoption**			
*Reasons for participating, indicated in the Reach Survey*			
Improve health	12	11	1
Education/desire for knowledge	2	2	0
*Reasons for not participating, indicated in the Reach Survey*			
Time constraints	24	20	4
Felt access would be an issue	4	2	2
Already active	4	2	2
Health or medical issues	2	2	0
Lack of motivation	1	1	0
Forgot to respond	1	1	0
Expressed initial interest, indicated through recruitment efforts	41	36	5
(% of total population)	(8.32)	(12.54)	(2.43)
Enrolled in the pilot program	25	24	1
(% of total population)	(5.07)	(8.36)	(0.49)
Did not enroll in the pilot program	16	12	4
(% of total population)	(3.25)	(4.18)	(1.94)
*Reasons for not entering program, indicated through recruitment efforts*			
Lost to follow-up (no reason specified)	6	4	2
Time constraints	3	3	0
Acute or chronic injury	2	1	1
Personal reasons	1	1	0
Retired	1	1	0
Refused to obtain medical clearance for a previous health condition	1	1	0
Didn’t want to focus on physical activity	1	0	1
Felt they could do it alone	1	1	0

Survey responses indicated that the preferred methods of recruitment were consistent with those methods that were used; however, recruitment could have benefited from more promotion over a longer period of time with additional notifications through staff memos and newsletters (where applicable). Recruitment of community-based workers may have benefited from SMS notifications. Some responses to the Reach Survey and non-participant interviews indicated a level of confusion around the program, with responses referring to other well-being initiatives that were implemented by the organization. Four residential survey respondents made reference to a different (unrelated nutritional) well-being initiative that had been implemented in residential sites when describing their reasons for choosing not to participate. This was supported by some level of confusion and uncertainty around the program that was seemingly present within the non-program participant interviews, where some interview participants were not sure if they were thinking of the correct program.

Sometimes I get a bit mixed up with other programs. We get a lot of information through work, and I just read things through in the moment.

Non-participant 02, community support worker

Through thematic analysis of qualitative interviews, all barriers to reach seemed to be related to issues of communicating the program information to potential participants. Barriers included workers missing or not reading information due to having a relatively large number of email communications and the automatic deletion of emails. Promotion of the program using a face-to-face approach (i.e., at team meetings) and promotion via team leaders or line managers were identified as factors that did facilitate, or could have improved, the reach of the program.

I don’t know whether it’s practical for someone like yourself, or your team, or – I’m not quite sure how the project is run – to be at every one of those team meetings. But one of our team leaders is definitely at one of those meetings. So that’s definitely a much better way to get the message across. We’re in a small group. We take it in better, and they’re really mandatory. We have to be there.

Non-participant 01, community-based worker

Recruitment for the pilot program took place over a 6-month period (October 2017–May 2018). The final number of participants adopted into the pilot program included 5.07% of the invited population. The median age of the program sample was 57 years, which was slightly older but relatively comparable with the median age of the whole community-based employee population (52 years). The sample was 88% female compared with 85% of the total community-based population. Demographic information for the total residential worker population was not available for comparison.

The interviews explored factors impacting adoption into the program. Barriers to adoption included competing priorities, cognitive factors, and current physical activity levels. Consistent with the pre-intervention interviews and Reach Survey responses, competing priorities that acted as barriers to adoption to the program consisted of home and family commitments, and associated work–life balance and lack of time.

And again, I don’t have time - I just wish I had time for me. The only time I have off, when the kids go to bed and I watch my shows, and then I have to go to sleep. So I just - I do try and focus on the weekends catching up with friends at night-time with them coming over, in between the sports during the day, et cetera. And that’s my “me time” to say, “okay, I’m seeing the girls,” or, “the family’s coming over,” or we’re visiting somebody else or going to a birthday party. So yeah, that’s it.

Non-participant 03, community-based worker

Other participants reported to be maintaining regular physical activity, either through work or in leisure time, as a reason for not participating in the program. Cognitive factors included wanting to keep their work and private lives separate and a reluctance to enter into the program if they felt that they would not be able to commit to it entirely.

If I can’t make a commitment to something, I can’t - I’m not going to do it because I won’t be able to put in 100%, but I didn’t want to waste your time doing that when I’m not going to do it, you know?

Non-participant 03, community-based worker

Potential facilitators of adoption from the interviews included factors relating to the nature of the program; nature of the promotion; and having site level input, involvement, and support. Factors relating to the nature of the program included having the choice to engage without pressure, keeping the program/information relevant to the workers, and offering initiatives at set or suitable times. Having the choice to engage without pressure mainly encompassed positive feedback around having other well-being services, or potential services such as the Activity for Well-Being program, as something that staff have the option to access if they want to but were not obligated to do so.

I’ve never actually – I’ve worked in lots of different industries in my life and I’d probably say this is the first industry that I’ve worked in that offers like all sorts of different supports, networks, to their employees. So even if you –whether you choose to use them or to access them or not, that’s to your discretion. But at least we’re always made aware that they are there if we need them.

Non-participant 04, community support worker

The qualitative information from the interview data was consistent with the findings of the Reach and Implementer Surveys. Interviews indicated that using a face-to-face approach and having clearer communication about the nature of the program were potential facilitators relating to adoption of the program. Other potential facilitators were selling the program better and including multiple prompts (nature of the promotion). Having site level input, involvement, and support was also identified as a strong theme throughout the interviews. In accord with factors influencing the reach of the study, strategies such as making team leaders a part of the process, having better input at the local/site level, and having support from those around the workers (such as line managers and work peers) were thought to be factors that could improve participation in the program at the individual level.

We would’ve been a good resource for you because in a lot of cases, we’ve known these people for a long time and there’s that trust element. So, often, if I have to read through a lot of information myself or do something, but if someone who I really trust says to me, “this is a great thing, you should just try it,” you just go, okay, I’ll give that a try.

Implementer staff 01, community

But we just think yeah…not realizing how much it could actually benefit us as individuals. Because, even I’m sort of thinking now, well I’m learning more talking to you now than I knew before, so yeah, I don’t know how else you’d sort of bring things to people’s fore. Unless you try and got somebody like [us] that could bring things, a little bit more information to staff and then be a bit more of an encourager.

Implementer staff 02, residential

Findings of the Implementer Survey highlighted a notably better understanding of the program by the community-based implementers compared with those in residential sites. The most frequently stated perceived barriers to adoption from the perspective of the implementing staff were reported as time constraints, a lack of confidence in individuals’ ability to be active, and a perceived lack of support from those around them (i.e., friends/family/work peers). A potential lack of interest in the program itself was also highlighted by two of the residential-based implementers. Despite this, five out of the six implementing staff felt that it would be valuable to maintain the use of a program such as this in the future, with the remaining staff member suggesting the use of a well-being initiative, which becomes part of the training agenda and is partially funded.

#### Fidelity

Assessment of the fidelity recordings demonstrated high fidelity for all aspects of education around the program components and for the collaborative development of the program for all recorded interviews. The fundamental principles of motivational interviewing were assessed as having high fidelity for the use of an empathetic approach, rolling with resistance, supporting self-efficacy, and supporting autonomy; the support and development of discrepancy by the AEP could have been improved in the recorded interviews. In addition to the assessment of the fidelity recordings, the results of the HCCQ demonstrated high levels of autonomy support. From 25 program participants, the HCCQ had 16 respondents and achieved a mean score of 6.61 out of 7 (±0.54). Good fidelity of the implementation of the autonomy-supportive approach through the initial appointments and across the course of the intervention and follow-up was also supported by the qualitative data from the interviews (see *Program Components* and table of selected quotes in Supplementary Material – program components).

#### Context

Themes from the interview data that related to context included work culture, work population (individual factors within the work context), and work structure (nature of the work). Some differences in work culture appeared to be present in the community settings compared with residential contexts. Positive relationships and trust between community-based workers and their team leaders were outlined by community-based implementers, program participants, and non-program participants. Particularly important for this seemed to be a team leader understanding and experience of the role of the frontline workers as well as close communications between the two.

What also is really good about our team leaders is our team leaders used to do our job, and previously the rostering staff used to do our job, and the advisors used to do our job. And anyone that used to do the job of the people that they’re now in charge of is so much better at their job.

Non-participant 01, community support worker

Short and long-term changes within the structure of the work also impacted work stress and culture. Substantial changes to work structure in the short-term that were occurring around the time of the 9-month measures appeared to have a large impact on work stress within the community-based population. Longer-term changes included community-based employees moving from a physical space and face-to-face interaction with work peers and line managers to an almost exclusive interaction via mobile devices due to changes in technology. This latter change seemed to have some impact on social support within a workplace context.

We all used to work out of this office…and it was a lovely time because you’d always be popping in there to get out your rosters and things and you’d run into people. You’d run into the office staff and it was a really lovely atmosphere and that’s all gone now.

Program participant 10, community support worker

Within residential settings, work stresses were focused more around staffing and residents (i.e., the stress of dealing with residents passing away or behavioral problems). As outlined in the barriers to adoption, there also appeared to be a preference to keeping work and personal lives separate, or a general hesitancy toward engaging with activities that were not directly a part of their work. In addition to these issues, physical workload as an element of work structure was identified as a source of fatigue and a barrier to participation for residential staff. This was consistent with the findings of the pre-program needs assessment undertaken with community-based workers.

We work all day, and it’s a physically demanding day. We just don’t want to do any more physically demanding things…but there’s some days that we just come home exhausted from work, and to actually think about doing more physical work – it would be a drain to even think about it.

Non-participant 05, residential care worker

### Intervention Mapping Evaluation

#### Performance and Change Objectives

Twenty-two program participants completed the 3-month questionnaire for the assessment of performance and change objectives. The mean participant-rated scores for each of the performance and change objectives are outlined in Supplementary Material 3. Most performance and change objectives achieved a positive change score (≥3.0). The performance objective to achieve the strongest mean change score was that for *identify opportunities to undertake physical activity* (4.05 ± 1.13). The remaining two performance objectives (*find time to undertake physical activity* and *find motivation to undertake physical activity*) received change scores of 3.73 (±1.24) and 3.93 (±1.13), respectively.

The final groupings of questions relating to the change objectives included three questions each in the autonomy and competence clusters and two questions in the relatedness cluster. One question (*do you feel that you are now more confident in your ability to control activity intensity?*) was initially considered to include elements of both autonomy and competence; however, the question had a poor fit with both clusters and was subsequently excluded from both of these clusters and presented as a stand-alone item. The composite scores for the autonomy, competence, and relatedness clusters were 4.16 (±0.88; α = 0.87), 3.75 (±1.24; α = 0.94), and 3.64 (±1.25; α = 0.84), respectively. The change score for the stand-alone item of *do you feel that you are now more confident in your ability to control activity intensity* was 3.90 (±1.26).

### Feasibility

Elements of the qualitative interviews relating to adherence, program components, subjective outcomes, and sustainability were used to inform the feasibility part of the study.

#### Adherence and Subjective Outcomes

Barriers to adherence as outlined within the post-program interviews were similar to those for adoption and those outlined within the pre-program interviews. These included competing priorities (i.e., home and family commitments, and time constraints); environmental and accessibility issues (i.e., poor weather and accessibility of exercise facilities, including location and available times); and individual factors (i.e., fatigue and energy levels; injury and illness; and amotivation and resistance to change). One notable individual factor that was identified as a barrier to adherence, but was not noted within the pre-program interviews, was “not wanting to inconvenience people.” This barrier related to participants within the program not wanting to ask too much of the supporting AEP. One specific example of this was a change of gyms for one participant (due to convenience of location). The program AEP supported the participant with adapting her initial program and using the equipment in the new gym; however, the participant was initially reluctant ask for this support since the AEP had also assisted the participant with the previous gym.

Because I’d gotten used to the [first gym], and then I thought “oh, this [second gym],” there’s no-one there; can’t get anyone to help you. And I think I’d joined up on the “I’m going to do it” because I didn’t want to lose that continuity of I’m going to stop again. And the whole thought, went in there and gone “oh, how am I going to do all this again, is it going to be the same equipment?” or whatever, and I’d sort of temporarily lost the mojo for a while of going “yeah, I don’t know.” And so then you sort of offered to do that and I’m like “oh”…“am I asking too much for that to happen?”

Program participant 25, community support worker

Facilitators of adherence included having accessible and comfortable environments, overcoming barriers to physical activity, personal motivators, social factors and support, and taking charge (independence) with behavior. The closely related sub-themes of taking charge (independence) with behavior and overcoming barriers to activity indicated a level of resilience in some of the program participants who appeared to facilitate adherence to regular physical activity participation.

I might have adapted something differently at different times. When I’ve stopped going to [the gym], so I’ve tried to sort of compensate and do things.

Program participant 20, community support worker

Subjective outcomes of the program described within the interviews with program participants were varied but generally positive. These included subjective improvements in mental and physical health, improved motivation and mindset, positive emotion (i.e., pride, sense of achievement, and feeling of value), and positive changes in perceptions around physical activity and health behavior. Many of these subjective outcomes appeared to be somewhat interrelated with participants expressing concurrent improvements in different subjective outcomes (i.e., improvements in physical and mental health; and improvements in mental health and motivation).

But what happened was doing this program, is this program actually…helped me reach my – it was my mental senses, you know my state of mental health and my sense of – it motivated me. So it motivated me to be more active about everything and one of the problems that I’ve had with this depression and anxiety thing, is pursuing the things that I love.

Program participant 01, community support worker

I can see the benefits of what it’s done for me improving my mental health and physical health. I sleep better and I’m enjoying life a lot better. It’s definitely a plus. I can see the relationship between the exercise and mental health; helping to improve it.

Program participant 09, voluntary community worker

Positive changes in perceptions around physical activity and health behavior included changes such as increased awareness of habits and decreased negative emotions around relapse.

I think I generally do quite well but every now and then I fall off the wagon, as I have with my eating. That’s the thing that starts it and I’ve just come to terms with it, “Well, we all do that. It’s not the end of the world.” I’ve learnt not to be so hard on myself. I think that’s it. I get very hard on myself.

Program participant 10, community support worker

Similarly, positive emotions included the expression of positive emotions such as pride, a sense of achievement, and a feeling of value.

But I guess it’s just mainly the satisfaction of knowing that I have done this myself – I mean with your help – and I am going to stick to it. Yeah. I am going to stick to it, and there will be times that I will probably lapse, and there’s probably times that I will do more but I know that it’s all there and it’s available to me.

Program participant 15, community support worker

#### Program Components

The use of self-monitoring of step counts or other health behaviors (via a pedometer or other wearable device, diary, or activity calendar) was strongly endorsed by participants throughout the interviews as a way of managing their own behavior. Setting or using goals, exercise programming, and technical support from the AEP were also strongly identified as components of the program that assisted participants to manage their activity levels and develop independence with behavior management.

Well I’m setting myself goals. I’m doing that and it’s just got me thinking. It’s always in the back of my head now. Always in there and yeah, it’s hard to get out what I’m trying to say. Definitely it has; it’s given me the motivation, and I have started thinking outside the square too and it’s got me thinking about more ways that I can try and do things and make it fun.

Program participant 04, community support worker

Yeah, a lot of structure to my exercise routine, like the gym and that sort of thing. Going in there and being shown machines to use for what I was trying to achieve and that sort of thing. So yeah, it’s given a lot of structure to that, and encouragement to continue and go for it.

Program participant 09, voluntary community worker

In addition to the self-management tools, the need support from the AEP was strongly referenced as a program component that was positively viewed by program participants. Of the sub-theme need support and affect, having someone that was non-judgmental and the participants felt comfortable contacting was viewed particularly positively.

Well, the program has helped me tremendously and having you helped because I know I can ring you up at any time. That’s been great. And yeah, just the whole aspect of it. I feel as if someone is prepared to listen to me and cares.

Program participant 15, community support worker

You haven’t judged us, whether we’ve done it or not, you haven’t said “oh, you’re a rotten person, you should be up to doing five days a week by now, at least 5 km every time you go out.” You haven’t done that to us, and I think that’s important as well.

Program participant 13, community support worker

Other program components had mixed responses. Newsletters were generally well accepted, and positive feedback centered around the newsletters offering small amounts of information without extensive reading. The website had only a small number of regular users (12% of program participants regularly using the website for tracking step counts and 12% using the website for a short time before ceasing to log in), with a large number of participants who never used the website (76% of program participants). The two primary reasons for this were forgetting about the website and not being “tech savvy.”

#### Sustainability

Although sustainability was not formally addressed within the pilot trial or evaluation of the Activity for Well-Being project, naturally emerging discussion within the interviews addressed some aspects of sustainability. Across the post-program interviews, several of the interview participants spoke positively about a number of ongoing well-being services that the organization had in place (i.e., skin checks, influenza vaccinations, confidential counseling, and early intervention physiotherapy). Conversely, some other short-term initiatives seemed to be met with criticism and a lack of engagement. Responses from program participants, non-participants, and implementing staff indicated a good level of support for incorporating a similar program into the organization on a long-term basis.

The primary reasons for program participants in the current study not utilizing organizational gym facilities (despite 20% of program participants choosing to utilize the university gyms during the course of the program) were all related to the accessibility of the facilities. Most organizational gyms were only available for a very limited number of hours per day (those where the site was accessible but the gym was not being used for customer services), which generally overlapped with participant working hours.

Yeah and I did ask about that [the gym] but unless it’s during working hours, and now they’ve been promoting more they’re getting, the gym it’s quite full…And I mean they opened up the great big new gym complex down, that’s [in one area], which isn’t feasible for me distance wise because I live [in another]. But once again for us to use the equipment after hours - that’s not, it’s a no go.

Program participant 20, community support worker

In some areas, organizational gyms not being available in the local area or difficulties finding time to be inducted into the gym were also barriers to utilizing these facilities.

One of the nurses has been trying to do an induction for I don’t know how long, they keep saying, “I will… do it”, but she wants to do it in five minutes… so, that’s sort of put her off.

Implementing staff 02, residential

## Discussion

Process evaluation of the pilot trial of the Activity for Well-Being program indicated moderate reach, with just over half of the community-based workers and a slightly higher proportion of the residential workers who completed the Reach Survey reporting that they were aware of the program. Despite this, there was poor adoption into the pilot trial, with only 5% of the whole population choosing to participate. This is compared with the 27–84% reported by a previous review of participation in workplace exercise/fitness programs (Glasgow et al., 1993) and previous trials with aged care employees that have ranged between 49 and 61% (Gerdle et al., 1995; Brox and Frøystein, 2005; Christensen et al., 2012). The most common reasons for participating in the pilot of the Activity for Well-Being program were related to a desire to improve health, while the most common reason for not participating was related to time constraints. These findings are consistent with previous studies such as the Step It Up Challenge, a workplace physical activity intervention in New Zealand, that reported the most prevalent reasons for participating in the program were associated with a desire to improve health and fitness (Davey et al., 2009); and the Healthy@Work program, a Tasmanian study that found time- and health-related factors were associated with lower levels of participation (Kilpatrick et al., 2017).

Qualitative and survey data from the current project indicated that the actual modes of recruitment used (i.e., emails, flyers, and presentations at team meetings) were appropriate; however, there was a need for more promotion through multiple sources, including promotion over a longer period of time and better education around the nature of the program, particularly in residential settings. Qualitative findings of the Activity for Well-Being project also indicated that incorporating local implementing staff (such as team leaders, line managers, or healthy aging staff) more actively into the project, as well as improving education about the program, may have improved employee engagement with the pilot program. More specifically, findings indicated the greater use of a face-to-face approach, directly or through line management, may have improved reach and adoption for the program across both community and residential settings and could have improved local knowledge around the program. An Austrian study by Nöhammer et al. (2010) noted that having easy and regular access to information and being personally notified may increase employee participation in workplace health promotion. Other studies have shown that perceived management support for such programs contributes to employee participation (Sloan and Gruman, 1988; Kilpatrick et al., 2017). Similarly, management support has previously been identified as impacting the implementation process for worksite health promotion programs (Wierenga et al., 2013). Within 36% of the primary studies included in a systematic review by Wierenga et al. (2013), poor management support was found to be a barrier to implementation, while strong management support was described as a facilitator of the implementation process. In the case of the Activity for Well-Being program, better understanding of the program by line management and more direct involvement could have improved managerial support and better facilitated the implementation process. In addition to these implementation issues, contextual factors could have played a role in the low adoption rates for the Activity for Well-Being project. One Danish study showed that low social support, fatiguing work, high physical demands/low job control, and high emotional demands/low job control were associated with low participation rates in workplace health promotion activities (Jørgensen et al., 2016). Factors such as these may be common for frontline aged care workers (Rao Hill and Clarke, 2010; Miranda et al., 2015) and potentially could have contributed to low levels of participation in the program.

Despite the low levels of adoption, positive feedback was received from those who participated in the program. Interview data from program participants indicated that the fundamental approach of the Activity for Well-Being program may be feasible for community-based frontline aged care workers. The fidelity of the autonomy-supportive approach of the program was confirmed by multiple datasets, including the fidelity recordings, the HCCQ scores, and the change scores for the autonomy cluster of the change objective questions.

Relatively high retention of participants within the Activity for Well-Being program and the positive subjective outcomes (health related, motivational, and behavioral) that were outlined in the interviews indicate good feasibility of the program for those who did choose to participate. Various components of the program were commented on during the interviews. Monitoring of step counts using a pedometer or other wearable device along with setting or using goals, exercise programming and technical support from the AEP, and having a support person who could be contacted (even if they did not need to be) were all program components that seemed to elicit the strong positive feedback from program participants. A large proportion of program participants self-reported frequent use of the Feeling Scale, RPE, or self-pacing for regulating activity intensity at 3 and 9 months. Coupling this with positive qualitative feedback around these self-determined methods of regulating physical activity intensity indicated that these could be a feasible and need-supportive strategy for exercise prescription within this population. Conversely, the website had mixed responses, and only a small number of program participants opted to use the website for the whole 9-month period. Improving the accessibility of organizational fitness facilities for the workers would also improve the feasibility of that aspect of the Activity for Well-Being program. Previous investigations into the preferences of different populations around the use of web-based health promotion have indicated some characteristic differences regarding preferences for mode of delivery. A study by Short et al. (2014) indicated that females and people with higher levels of physical activity tended to prefer face to face rather than print or online mediated interventions. Balk-Møller et al. (2017) had high levels of attrition and low numbers of active users in a web-based health promotion intervention in social welfare and health care sector employees, including some with very limited skills with smartphone technology. Conversely a study by Cook et al. (2015) showed good retention rates for a web-based program in a group of older office-based employees. One systematic review by Hobbs et al. (2013) suggested that mode of delivery does not appear to be important for effectiveness of behavioral interventions in adults aged 55–70 years; however, the review was not workplace specific and did not account for differences in population characteristics in regard to efficacy or participant preferences. While it is not entirely clear why some participants may prefer certain modes of delivery over others, the web-based component of the Activity for Well-Being program did not appear to be feasible (or preferable) for this particular cohort.

Barriers to adherence for program participants were found to be similar to those identified in the pre-program interviews (Lock et al., 2018), with the most prominent barrier being time constraints. Time constraints have long been established as one of the most prevalent barriers to regular physical activity participation in adults in both by cross-sectional and experimental studies (Booth et al., 1997; Trost et al., 2002; Mailey et al., 2014; Kilpatrick et al., 2017). One barrier that was specific to the Activity for Well-Being program was the issue of participants not wanting to inconvenience the supporting AEP, despite the scheduled follow-ups from the AEP during the active intervention period and the emphasis that participants could approach the AEP for any support at any stage throughout the intervention and follow-up. Strategies to address this more formally may be needed to decrease this as a barrier within future versions of the program. Considering this as a barrier to adherence, and acknowledging a need for workers to be able to access well-being services on their terms without pressure (considering the sub-theme of nature of the program), the Activity for Well-Being program may become more feasible if it was to be built into the organization at a broader level like an early intervention service, but incorporating local implementers, and could be accessed by staff without pressure or excessive perceived commitment. It is likely that the cost-effectiveness of this approach would be low unless the organization utilized resources (i.e., wellness staff and organizational gyms/facilities) that were already in place. Building well-being education into paid staff training was another potential strategy highlighted by program participants, non-program participants, and implementing staff and could address some of these issues.

The process evaluation highlighted that certain factors associated with the current delivery of the program would need to be addressed prior to implementing the intervention on a broader scale. These include implementation and contextual factors contributing to low adoption of the program, and thereby the cost-effectiveness of any adaptions of the program that may be developed in the future. Despite the inclusion of participatory, pre-program interviews, the findings of the process evaluation indicated a greater need for input and support at the local and site levels across all stages of the project. Input and support from line managers and team leaders in the early stages of the implementation were lacking in the current pilot of the program and may have improved both reach and adoption. This may be particularly important since the program was implemented in a large and geographically disperse organization and having more direct involvement of local staff and line management could potentially have facilitated a better understanding of the program and greater advocacy within trusted networks. Despite this, feedback from both community- and residential-based implementing staff indicated a good level of perceived value in continuing a program such as this on a broader scale within the organization.

It should also be noted that despite the positive feedback from those program participants who consented to an interview, there are several limitations of the current study. Firstly, the low levels of adoption into the pilot program may limit the ability to generalize the results to the whole target population and may have increased the risk of selection bias in the sample. Additionally, feedback could not be obtained from the few program participants who withdrew from the program. There was an under-representation of residential staff within the pilot program, which makes it all the more difficult to draw any conclusions regarding the feasibility of this type of program for residential workers.

## Conclusion

Process evaluation of the Activity for Well-Being program found that the reach of the program was moderate but adoption was low. The process evaluation of the Activity for Well-Being project also demonstrated clear differences between community and residential employee populations in regard to reach, adoption, and context. Recruitment for the pilot trial of the program initially encountered challenges resulting in a low adoption level into the program. This number was sufficient for the pilot trial; however, barriers to adoption would need to be addressed prior to implementing a full trial. General feedback from those that did participate in the program was positive, and retention of participants was high. The use of self-management tools and self-determined methods of regulating physical activity intensity appeared to be feasible, while other components of the program, such as the website, had mixed responses and low engagement. The simple element of having someone that participants could contact if they needed (even if they did not) elicited a strong positive response in the program participant interviews. Qualitative information also indicated that involving local implementing staff, such as team leaders or line managers, more directly into the intervention could have potentially improved reach, adoption, and feasibility of the program.

## Data Availability Statement

The data that support the conclusions of this article will be made available on request by the corresponding author. The data are not publicly available due to content that could compromise the privacy of research participants.

## Ethics Statement

All parts of this study involving human participants were reviewed and approved by the Human Research Ethics Committee, University of South Australia. All participants provided their written and/or verbal informed consent to participate in the pilot study and process evaluation.

## Author Contributions

ML undertook initial development of this manuscript with all authors having contributed the final drafts of the manuscript. ML and DP collaborated on the development of the evaluation plan, the change and performance objectives matrices, and qualitative analyses. JD and GP have overseen the entire project from initiation to present and have contributed to the research design and implementation of all aspects of the plan outlined within this manuscript. All authors contributed to the article and approved the submitted version.

## Conflict of Interest

The authors declare that the research was conducted in the absence of any commercial or financial relationships that could be construed as a potential conflict of interest.

## References

[B1] Abraham C. Graham-Rowe E. (2009). Are worksite interventions effective in increasing physical activity? A systematic review and meta-analysis. *Health Psychol. Rev.* 3 108–144. 10.1080/17437190903151096

[B2] Balk-Møller N. C. Larsen T. M. Holm L. (2017). Experiences from a web- and app-based workplace health promotion intervention among employees in the social and health care sector based on use-data and qualitative interviews. *J. Med. Internet Res.* 19:e350. 10.2196/jmir.7278 29051133 PMC5668633

[B3] Bartholomew L. K. Parcel G. S. Kok G. (1998). Intervention mapping: a process for developing theory- and evidence-based health education programs. *Health Educ. Behav.* 25 545–563. 10.1177/109019819802500502 9768376

[B4] Bartholomew L. K. Parcel G. S. Kok G. Gottlieb N. H. Fernández M. E. (2011). *Planning health Promotion Programs: an Intervention Mapping approach*, 3rd Edn, Hoboken, NJ: John Wiley & Sons.

[B5] Booth M. L. Bauman A. E. Owen N. Gore C. J. (1997). Physical activity preferences, preferred sources of assistance, and perceived barriers to increased activity among physically inactive Australians. *Prevent. Med.* 26 131–137. 10.1006/pmed.1996.9982 9010908

[B6] Borg G. A. V. (1982). Psychophysical bases of perceived exertion. *Med. Sci. Sports Exerc.* 14 377–381. 7154893

[B7] Braun V. Clarke V. (2013). *Successful Qualitative Research: A Practical Guide for Beginners.* London: Sage Publications.

[B8] Brox J. I. Frøystein O. (2005). Health-related quality of life and sickness absence in community nursing home employees: randomized controlled trial of physical exercise. *Occup. Med.* 55 558–563. 10.1093/occmed/kqi153 16251373

[B9] Brug J. Oenema A. Ferreira I. (2005). Theory, evidence and Intervention Mapping to improve behavior nutrition and physical activity interventions. *Intern. J. Behav. Nutr. Phys. Activ.* 2 1–7. 10.1186/1479-5868-2-2 PMC1087867 15807898

[B10] Christensen J. R. Overgaard K. Carneiro I. G. Holtermann A. Søgaard K. (2012). Weight loss among female health care workers- a 1-year workplace based randomized controlled trial in the FINALE-health study. *BMC Public Health* 12:625. 10.1186/1471-2458-12-625 22871173 PMC3487739

[B11] Cook R. F. Hersch R. K. Schlossberg D. Leaf S. L. (2015). A web-based health promotion program for older workers: randomized controlled trial. *J. Med. Internet Res.* 17:e82. 10.2196/jmir.3399 25830503 PMC4390614

[B12] Craig P. Dieppe P. Macintyre S. Michie S. Nazareth I. Petticrew M. (2008). developing and evaluating complex interventions: the new medical research council guidance. *Br. Med. J.* 337:1655. 10.1136/bmj.a1655 PMC2769032 18824488

[B13] Davey J. Fitzpatrick M. Garland R. Kilgour M. (2009). Adult participation motives: empirical evidence from a workplace exercise program. *Eur. Sport Manag. Q.* 9 141–162. 10.1080/16184740802571427

[B14] Deci E. L. Ryan R. M. (2000). The ‘what’ and ‘why’ of goal pursuits: human needs and the self-determination of behavior. *Psychol. Inq.* 11 227–268. 10.1207/s15327965pli1104_01

[B15] Dombrowski S. U. Sniehotta F. F. Avenell A. (2007). Current issues and future directions in psychology and health : towards a cumulative science of behaviour change: do current conduct and reporting of behavioural interventions fall short of best practice? *Psychol. Health* 22 869–874. 10.1080/08870440701520973

[B16] Dugdill L. Brettle A. Hulme C. McCluskey S. Long A. (2008). Workplace physical activity interventions: a systematic review. *Intern. J. Workplace Health Manag.* 1 20–40. 10.1108/17538350810865578

[B17] Edmunds J. Ntoumanis N. Duda J. L. (2006). A test of self-determination theory in the exercise domain. *J. Appl. Soc. Psychol.* 36 2240–2265. 10.1111/j.0021-9029.2006.00102.x

[B18] Edmunds J. Ntoumanis N. Duda J. L. (2008). Testing a self-determination theory-based teaching style intervention in the exercise domain. *Eur. J. Soc. Psychol.* 38 375–388. 10.1002/ejsp.463

[B19] Fortier M. S. Sweet S. N. O’Sullivan T. L. Williams G. C. (2007). A self-determination process model of physical activity adoption in the context of a randomized controlled trial. *Psychol. Sport Exerc.* 8 741–757. 10.1016/j.psychsport.2006.10.006

[B20] Frensham L. J. Zarnowiecki D. M. Parfitt G. King S. Dollman J. (2014). The experiences of participants in an innovative online resource designed to increase regular walking among rural cancer survivors: a qualitative pilot feasibility study. *Support. Care Cancer* 22 1923–1929. 10.1007/s00520-014-2177-4 24573604

[B21] Friederichs S. A. H. Oenema A. Bolman C. Lechner L. (2016). Motivational Interviewing and self-determination theory in a web-based computer tailored physical activity intervention: a randomized controlled trial. *Psychol. Health* 31 1–24. 26849996 10.1080/08870446.2016.1151018

[B22] Gerdle B. Brulin C. Elert J. Eliasson P. Granlund B. (1995). Effect of a general fitness program on musculoskeletal symptoms, clinical status, physiological capacity, and perceived work environment among home care service personnel. *J. Occup. Rehabil.* 5 1–16. 10.1007/bf02117816 24469864

[B23] Glasgow R. E. Harden S. M. Gaglio B. Rabin B. Smith M. L. Porter G. C. (2019). RE-AIM planning and evaluation framework: adapting to new science and practice with a 20-year review. *Front. Public Health* 7:64. 10.3389/fpubh.2018.00064 30984733 PMC6450067

[B24] Glasgow R. E. McCaul K. D. Fisher K. J. (1993). Participation in worksite health promotion: a critique of the literature and recommendations for future practice. *Health Educ. Q.* 20 391–408. 10.1177/109019819302000309 8307762

[B25] Glasgow R. E. Vogt T. M. Boles S. M. (1999). Evaluating the public health impact of health promotion interventions: the RE-AIM framework. *Am. J. Public Health* 89 1322–1327. 10.2105/ajph.89.9.1322 10474547 PMC1508772

[B26] Greaves C. J. Sheppard K. E. Abraham C. Hardeman W. Roden M. Evans P. H. (2011). Systematic review of reviews of intervention components associated with increased effectiveness in dietary and physical activity interventions. *BMC Public Health* 11:119. 10.1186/1471-2458-11-119 21333011 PMC3048531

[B27] Hamlyn-Williams C. C. Tempest G. Coombs S. Parfitt G. (2015). Can previously sedentary females use the feeling scale to regulate exercise intensity in a gym environment? an observational study. *BMC Sports Sci. Med. Rehabil.* 7:30. 10.1186/1471-2458-13-30 26613045 PMC4660653

[B28] Hardy C. J. Rejeski W. J. (1989). Not what, but how one feels: the measurement of affect during exercise. *J. Sport Exerc. Psychol.* 11 304–317. 10.1123/jsep.11.3.304

[B29] Hobbs N. Godfrey A. Lara J. Errington L. Meyer T. D. Rochester L. (2013). Are behavioral interventions effective in increasing physical activity at 12 to 36 months in adults aged 55 to 70 years? a systematic review and meta-analysis. *BMC Med.* 11:75. 10.1186/1471-2458-13-75 23506544 PMC3681560

[B30] Hoddinott P. (2015). A new era for intervention development studies. *Pilot Feasibil. Stud.* 1:36. 10.1186/s40814-015-0032-0 PMC5153779 27965814

[B31] Issel L. M. Wells R. (2017). *Health Program Planning and Evaluation: A Practical, Systematic Approach for Community Health*, 4th Edn, Burlington, MA: Jones & Bartlett Learning LLC.

[B32] Jørgensen M. B. Villadsen E. Burr H. Punnett L. Holtermann A. (2016). Does employee participation in workplace health promotion depend on the working environment? A cross-sectional study of Danish workers. *BMJ Open* 6:e010516. 10.1136/bmjopen-2015-010516 27279474 PMC4908961

[B33] Kilpatrick M. Blizzard L. Sanderson K. Teale B. Jose K. Venn A. (2017). Barriers and facilitators to participation in workplace health promotion (WHP) activities: results from a cross-sectional survey of public-sector employees in Tasmania, Australia. *Health Promot. J. Austr.* 28 225–232. 10.1071/he16052 28110642

[B34] Kok G. Schaalma H. Ruiter R. A. C. Van Empelen P. Brug J. (2004). Intervention Mapping: Protocol for applying health psychology theory to prevention programmes. *J. Health Psychol.* 9 85–98. 10.1177/1359105304038379 14683571

[B35] Levy S. S. Cardinal B. J. (2004). Effects of a self-determination theory-based mail-mediated intervention on adults’ exercise behavior. *Am. J. Health Promot.* 18 345–349. 10.4278/0890-1171-18.5.345 15163133

[B36] Lock M. Post D. Dollman J. Parfitt G. (2018). Development of a self-determination theory-based physical activity intervention for aged care workers: protocol for the activity for well-being program. *Front. Public Health* 6:341. 10.3389/fpubh.2018.00341 30534548 PMC6275311

[B37] Mailey E. L. Huberty J. Dinkel D. McAuley E. (2014). Physical activity barriers and facilitators among working mothers and fathers. *BMC Public Health* 14:657. 10.1186/1471-2458-13-657 24974148 PMC4227023

[B38] Malik S. H. Blake H. Suggs L. S. (2013). A systematic review of workplace health promotion interventions for increasing physical activity. *Br. J. Health Psychol.* 19 149–180. 10.1111/bjhp.12052 23827053

[B39] Miller W. R. (1983). Motivational Interviewing with problem drinkers. *Behav. Psychother.* 11 147–172. 10.1017/s0141347300006583

[B40] Miranda H. Gore R. J. Boyer J. Nobrega S. Punnett L. (2015). Health behaviors and overweight in nursing home employees: contribution of workplace stressors and implications for worksite health promotion. *Sci. World J.* 2015:10. 10.1155/2015/915359 PMC4561990 26380373

[B41] Moore G. F. Audrey S. Barker M. Bond L. Bonell C. Hardeman W. (2015). Process evaluation of complex interventions: medical research council guidance. *Br. Med. J.* 350:h1258. 10.1136/bmj.h1258 25791983 PMC4366184

[B42] Ng J. Y. Y. Ntoumanis N. Thøgersen-Ntoumani C. Stott K. Hindle L. (2013). Predicting psychological needs and well-being of individuals engaging in weight management: the role of important others. *Appl. Psychol. Health Well Being* 5 291–310. 10.1111/aphw.12011 23713059

[B43] Nöhammer E. Schusterschitz C. Stummer H. (2010). Determinants of employee participation in workplace health promotion. *Intern. J. Workplace Health Manag.* 3 97–110. 10.1108/17538351011055005

[B44] O’Brien N. McDonald S. Araújo-Soares V. Lara J. Errington L. Godfrey A. (2015). The features of interventions associated with long-term effectiveness of physical activity interventions in adults aged 55-70 years: a systematic review and meta-analysis. *Health Psychol. Rev.* 9 417–433. 10.1080/17437199.2015.1012177 25689096

[B45] Painter J. E. Borba C. P. C. Hynes M. Mays D. Glanz K. (2008). The use of theory in health behavior research from 2000 to 2005: a systematic review. *Ann. Behav. Med.* 35:358. 10.1007/s12160-008-9042-y 18633685

[B46] Parfitt G. Rose E. A. Burgess W. M. (2006). The psychological and physiological responses of sedentary individuals to prescribed and preferred intensity exercise. *Br. J. Health Psychol.* 11 39–53. 10.1348/135910705x43606 16480554

[B47] Prestwich A. Sniehotta F. F. Whittington C. Dombrowski S. U. Rogers L. Michie S. (2014). Does theory influence the effectiveness of health behavior interventions? Meta-analysis. *Health Psychol.* 33 465–474. 10.1037/a0032853 23730717

[B48] Quested E. Ntoumanis N. Thøgersen-Ntoumani C. Hagger M. S. Hancox J. E. (2017). Evaluating quality of implementation in physical activity interventions based on theories of motivation: current challenges and future directions. *Intern. Rev. Sport Exerc. Psychol.* 10 252–269. 10.1080/1750984x.2016.1217342

[B49] Rao Hill S. Clarke M. (2010). Linking employee wellbeing and stakeholder quality of life: The case of aged care, *Paper Presented at ANZAM Conference*, Adelaide, SA.

[B50] Rhodes R. E. Kates A. (2015). Can the affective response to exercise predict future motives and physical activity behavior? A systematic review of published evidence. *Ann. Behav. Med.* 49 715–731. 10.1007/s12160-015-9704-5 25921307

[B51] Rollnick S. Allison J. (2004). “Motivational interviewing,” in *The Essential Handbook of Treatment and Prevention of Alcohol Problems*, eds Heather N. Stockwell T. (Chichester: John Wiley & Sons Ltd), 105 10.1093/med/9780190619954.003.0011

[B52] Rose E. A. Parfitt G. (2008). Can the feeling scale be used to regulate exercise intensity? *Med. Sci. Sports Exerc.* 40 1852–1860. 10.1249/mss.0b013e31817a8aea 18799997

[B53] Ryan R. M. Deci E. L. (2000). Self-determination theory and the facilitation of intrinsic motivation, social development, and well-being. *Am. Psychol.* 55 68–78. 10.1037/0003-066x.55.1.68 11392867

[B54] Shepperd S. Lewin S. Straus S. Clarke M. Eccles M. P. Fitzpatrick R. (2009). Can we systematically review studies that evaluate complex interventions? *PLoS Med.* 6:e1000086. 10.1371/journal.pmed.1000086 19668360 PMC2717209

[B55] Short C. E. Vandelanotte C. Duncan M. J. (2014). Individual characteristics associated with physical activity intervention delivery mode preferences among adults. *Intern. J. Behav. Nutr. Phys. Activ.* 11:25. 10.1186/1479-5868-11-25 24568611 PMC3938301

[B56] Sloan R. P. Gruman J. C. (1988). Participation in workplace health promotion programs: the contribution of health and organizational factors. *Health Educ. Q.* 15 269–288. 10.1177/109019818801500303 3192406

[B57] Taylor N. Conner M. Lawton R. (2012). The impact of theory on the effectiveness of worksite physical activity interventions: a meta-analysis and meta-regression. *Health Psychol. Rev.* 6 33–73. 10.1080/17437199.2010.533441

[B58] Trost S. G. Owen N. Bauman A. E. Sallis J. F. Brown W. (2002). Correlates of adults’ participation in physical activity: review and update. *Med. Sci. Sports Exerc.* 34 1996–2001. 10.1097/00005768-200212000-00020 12471307

[B59] Vazou-Ekkekakis S. Ekkekakis P. (2009). Affective consequences of imposing the intensity of physical activity: does the loss of perceived autonomy matter. *Hellen. J. Psychol.* 6 125–144.

[B60] Weman-Josefsson K. Lindwall M. Ivarsson A. (2015). Need satisfaction, motivational regulations and exercise: moderation and mediation effects. *Intern. J. Behavi. Nutr. Phys. Activ.* 12 1–11. 10.1186/s12966-015-0226-0 PMC4489042 25990492

[B61] Wierenga D. Engbers L. H. Van Empelen P. Duijts S. Hildebrandt V. H. Van Mechelen W. (2013). What is actually measured in process evaluations for worksite health promotion programs: a systematic review. *BMC Public Health* 13:1190. 10.1186/1471-2458-13-1190 24341605 PMC3890539

[B62] Williams D. M. (2008). Exercise, affect, and adherence: an integrated model and a case for self-paced exercise. *J. Sport Exerc. Psychol.* 30 471–496. 10.1123/jsep.30.5.471 18971508 PMC4222174

[B63] Williams D. M. Dunsiger S. Ciccolo J. T. Lewis B. A. Albrecht A. E. Marcus B. H. (2008). Acute affective response to a moderate-intensity exercise stimulus predicts physical activity participation 6 and 12 months later. *Psychol. Sport Exerc.* 9 231–245. 10.1016/j.psychsport.2007.04.002 18496608 PMC2390920

[B64] Williams G. C. Grow V. M. Freedman Z. R. Ryan R. M. Deci E. L. (1996). Motivational predictors of weight loss and weight-loss maintenance. *J. Pers. Soc. Psychol.* 70 115–126. 10.1037/0022-3514.70.1.115 8558405

