# Supplementary material for: Feasibility and Process Evaluation of a Need-Supportive Physical Activity Program in Aged Care Workers: The Activity for Well-Being Project
Source: Front Psychol. 2020 Sep 30;11:518413. doi: 10.3389/fpsyg.2020.518413 (PMC7554301; doi:10.3389/fpsyg.2020.518413)
Supplement: Supplementary file 1 [file Table_1.DOCX]

**Supplementary Material 1a** Performance objectives matrix

**`**

| **Health Outcome:** Physical health and psychological wellbeing in support workers | | | | |
| --- | --- | --- | --- | --- |
| **Behavioural Outcome of Participants:** Physical activity adoption and maintenance | | | | |
| **Organisational Outcome:** To increase awareness within the organisation regarding organisational factors that impact support worker engagement in physical activity | | | | |
|  | **Determinants** | | | |
| **Performance Objectives**  *(sub-behaviours of target behaviours)* | **Support for Autonomy** | **Support for Competence** | **Support for Relatedness** | **Positive Exercise Affect** |
| **Find time to undertake physical activity** | Choose activities that are flexible (can be undertaken in their own time) or build activity into their day | Express confidence in their ability to find time to be physically active, even during busy days | Utilise opportunities to be active while taking care of competing priorities (i.e. incorporating family into activities or incorporating activity into housework) | Utilise affect-regulated activity to assist with stress management during busy times |
| **Find motivation to undertake physical activity** | Choose activity types and intensities based on how they are feeling on a particular day | Schedule exercise when they are more like to have motivation and energy | Schedule activities with friends, family or co-workers to increase motivation to complete it; Schedule ‘active catch-ups’ | Choose activities that are more enjoyable or that elicit more positive exercise affect |
| **Identify opportunities to undertake physical activity** | Identify activities that they personally have the facilities and motivation to undertake | Identify activities that they have the confidence and ability to undertake | Identify supportive environments to undertake activity | Identify activities that are more enjoyable or more likely to elicit positive affective valence |

*Each of the answers in the boxes (unbolded) are ‘change objectives’ – these are put into another matrix below*

**Supplementary Material 1b** Change objectives matrix - *find time to undertake physical activity*

| **Performance Objective: Find time to undertake physical activity** | | | | | |
| --- | --- | --- | --- | --- | --- |
| **Personal Determinants** | **Methods** | **Parameter for use** | **Change Objectives** | **Applications** | **Explanation** |
| **Support for Autonomy** | Flexible activities included in person-centred activity plans. | Behaviour needs to be perceived as volitional and consistent with own values | Choose activities that are flexible (can be undertaken in their own time) or build activity into their day | Work with participant to find activities that could be flexible within their schedule and to develop strategies to assist them to undertake activity at flexible times. | Sign-posting participants to activities such as walking that can be easily undertaken at any time and place. As a part of their activity plans, strategies such as wearing sandshoes to work or keeping activity clothes in the car to allow them to undertake activity (i.e. go walking) during scheduled or unexpected breaks will improve their chances of doing so. |
| **Support for Competence** | Action and coping planning. | Needs to reinforce ability to achieve goals and change behaviour | Express confidence in their ability to find time to be physically active, even during busy days | Educate participants re: strategies for fitting PA into busy schedules and the use of action and coping planning to self- manage activity. | Action and coping planning can be used to schedule activity and plan strategies to overcome barriers to activity. Previous studies have found action and coping planning can increase exercise and barrier self-efficacy (perceived competence) which will thereby enable the participant to express confidence in their ability to find time to be active, even during busy days. |
| **Support for Relatedness** | Person-centred activity plan and educational newsletters | Needs to be empathetic and supportive of behaviour change; needs to foster the support of others around them | Utilise opportunities to be active while taking care of competing priorities | Teaching skills (i.e. incorporating family into activities or incorporating activity into housework) | Competing priorities of home and family created a significant barrier to activity as identified during the pre-intervention qualitative study, therefore identifying activities that could be undertaken while still undertaking these roles may improve their likelihood of undertaking activity while also supporting their need for relatedness. Examples could include incorporating family (spouses or children) into activities or incorporating activity into housework. |
| **Positive Exercise Affect** | Affect-regulated activity | Needs to promote a more positive and less negative experience associated with the behaviour | Utilise affect-regulated activity to assist with stress management during busy times | Educate participant in the use of affect-regulated activity to assist with stress management, including during busy times. | The use of activities such as walking, particularly in natural environments, was an important enabler to the maintenance of activity in the qualitative interviews. The use of green activity has been shown to improve psychological measures and could be useful as a stress management technique for support workers. The use of affect-regulation the activity intensity should help to reinforce this as a positive experience as assist with the maintenance of it as a behaviour, even during busy times. |

**Supplementary Material 1c** Change objectives matrix - *find motivation to undertake physical activity*

| **Performance Objective: Find motivation to undertake physical activity** | | | | | |
| --- | --- | --- | --- | --- | --- |
| **Personal Determinants** | **Methods** | **Parameter for use** | **Change Objectives** | **Applications** | **Explanation** |
| **Support for Autonomy** | Affect-regulation and use of the Feeling Scale and motivational interviewing | Behaviour needs to be perceived as volitional and consistent with own values | Choose activity types and intensities based on how they are feeling on a particular day | Educate participants on the use of the Feeling Scale to regulate activity intensity and to choose activities that elicit more positive affective valence. Discuss activities that they have previously enjoyed or been successful at during the motivational interview and build into person-centred plan if appropriate. Eliciting ‘change talk’ and the expression of personal motives for behaviour change through motivational interviewing. | Using the Feeling Scale (or self-pacing) to regulate activity to elicit positive affective valence should facilitate the movement along the behavioural regulations continuum (to be more autonomous) and improve the maintenance of volitional activity (via the exercise-affect-adherence pathway). This should support perceived autonomy through the self-regulation of the activity intensity and by facilitating more personal (autonomous) motives. Additionally, eliciting the expression of more personal motives for activity through the motivational interviewing process should assist the participant to realise these motives and become more internally regulated in their behaviour. |
| **Support for Competence** | Person-centred activity plans | Needs to reinforce ability to achieve goals and change behaviour | Schedule exercise when they are more like to have motivation and energy | Collaborate with the participant to identify days and times that the participant may be less fatigued (i.e. mornings or non-work-days) and therefore more motivated to undertake blocks of activity. | If participants are supported (and taught skills) to identify the best times and days to undertake physical activity when they are likely to be less fatigued or more motivated, this will assist to minimise the barriers to undertaking activity. |
| **Support for Relatedness** | Person-centred activity plans | Needs to be empathetic and supportive of behaviour change; needs to foster the support of others around them | Schedule activities with friends, family or co-workers to increase likelihood of completing it; Schedule ‘active catch-ups’ | Encourage participants to schedule activities with friends or family and use web-forums and newsletters to sign-post to community activities and instigate activities with co-workers. | Not only does evidence suggest that support for relatedness is particularly important in the adoption phase of physical activity behaviour change, but social support was also mentioned as an enabler to activity adoption and maintenance during the qualitative interviews. As support workers tend work in isolation from their co-workers, the use of ‘activities’ to catch up with fellow support workers was mentioned as a desired activity. Being ‘accountable’ to someone else (scheduling a time and needing to be there) and having the social engagement may be a motivation to undertake scheduled activities. |
| **Positive Exercise Affect** | Sign-posting to different activities | Needs to promote a more positive and less negative experience associated with the behaviour | Choose activities that are more enjoyable or that elicit more positive exercise affect | Sign-posting to a large variety of activities, including those within the community and those that can be undertaken in their own time (i.e. walking and home-based exercise) gives the participants the opportunity to choose different activities. | Having a choice of activities enables the participants to choose activities that they are more likely to enjoy or those that they have succeeded at in the past improving the likelihood of positive affective response from the participant and more autonomous forms of motivation for the activity. It also supports the psychological needs for autonomy, competence and relatedness thereby theoretically improving the affective response in itself. |

**Supplementary Material 1d** Change objectives matrix – *identify opportunities to undertake physical activity*

| **Performance Objective: Identify opportunities to undertake physical activity** | | | | | |
| --- | --- | --- | --- | --- | --- |
| **Personal Determinants** | **Methods** | **Parameter for use** | **Change Objectives** | **Applications** | **Explanation** |
| **Support for Autonomy** | Action / coping planning and tiered goals using the website | Behaviour needs to be perceived as volitional and consistent with own values | Identify activities that they personally have the facilities and motivation to undertake | Teaching skills to set realistic goals and action plans and to adjust these depending on situations that arise and using coping plans to overcome barriers to activity | Emphasising the use of realistic goals and action plans for the identification of suitable activity types (i.e. not choosing high-cost activities when finances are a significant barrier for a person) and intensity (i.e. using affect and RPE to monitor intensity of activity at a level that is comfortable for the participant) and volume (i.e. using tiered goals within the website to adjust daily goals based on how they are feeling and other factors that may impact the ability to obtain the goals). Using strategies to set realistic plans and goals should enable the participant to feel more competent in their ability to achieve them and facilitate more autonomous motivation for activity. |
| **Support for Competence** | Motivational interviewing | Needs to reinforce ability to achieve goals and change behaviour | Identify activities that they have the confidence and ability to undertake | Using motivational interviewing techniques to identify activities that participants may have been previously successful at or feel more competent in undertaking | It is well-established that exercise self-efficacy is increased with participation in exercise. Identifying activities that the participant may have previously undertaken and been successful with may improve their likelihood of undertaking the activity since their self-efficacy for that particular activity should be higher. |
| **Support for Relatedness** | Provision of access to organisation gyms, website and newsletters | Needs to be empathetic and supportive of behaviour change; needs to foster the support of others around them | Identify supportive environments to undertake activity | Provision of organisation gyms for use for the intervention participants. Support for identifying different community-based activities that may be perceived as supportive can be provided via the website and newsletters (in the form of sign-posting to community activities and encouragement for participants to try different things and choose activities that support them. | Participants in the pre-intervention qualitative study identified that feeling uncomfortable or self-conscious in commercial gyms was a barrier to gym-based activity). Having access to gyms that are low cost (free to use for organisation employees) and are comfortable to use (i.e. are owned and run by the organisation and are only used by organisation residents and employees) provides them with the option for gym-based activity that may be preferable to larger, commercial gyms in the community. |
| **Positive Exercise Affect** | Use of affect- or self-regulation for activity | Needs to promote a more positive and less negative experience associated with the behaviour | Identify activities that are more enjoyable or more likely to elicit positive affective valence | Encouraging the use of affect regulation and self-regulation for activity should enable the participant to identify which activities they experience more positively | The choice of activities that elicit more positive affective valence should positively reinforce the behaviour and should lead to better maintenance of activity |
