# Supplementary material for: Feasibility and Process Evaluation of a Need-Supportive Physical Activity Program in Aged Care Workers: The Activity for Well-Being Project
Source: Front Psychol. 2020 Sep 30;11:518413. doi: 10.3389/fpsyg.2020.518413 (PMC7554301; doi:10.3389/fpsyg.2020.518413)
Supplement: Supplementary file 2 [file Table_2.DOCX]

**Supplementary Material 2** Themes, sub-themes and selected quotes

1. Process evaluation components (reach, adoption, and context)

| **Overarching theme** | **Theme** | **Sub-theme** | **Selected quote (s)** |
| --- | --- | --- | --- |
| **Reach** | **Barriers to reach** | **Communication issues** | *“Well, I think - I don't know how you would do it, but okay, we get a staff newsletter every week, but it's just a whole heap of them are put in the office on the desk. Okay? So, it's really up to the individual whether they see them, whether they want to read them, so I think that is a huge problem, and I don’t know how well - I mean, you can't force someone to read something. So, I mean, I think that is a problem in getting information out there, is the fact that, okay, yeah, there's a heap of newsletter put on the desk in the office. That’s it. So, if this research, for instance, was put in the newsletter, and maybe half a dozen people read it and the rest didn't, then you're not going to get your information out there, are you?”*  NP06, non-participant, residential care worker  *“I don’t know whether it’s practical for someone like yourself, or your team, or – I’m not quite sure how the project is run – to be at every one of those team meetings. But one of our team leaders is definitely at one of those meetings. So that’s definitely a much better way to get the message across. We’re in a small group. We take it in better, and they’re really mandatory. We have to be there.*  NP01, non-participant, community support worker  *“I’m not sure if this has changed, but up until now, the email system has been like this abhorrent thing, where everything that’s been in your mailbox for a month, whether it’s your sent mailbox or your inbox, just automatically deletes…I know I had a few emails that I was drafting, and I’d saved them in my draft box, and with the swap over of the phones, I even lost what I’d saved in my drafts section. So, yes, unless you’re really proactive about going through and reading your emails, if you think about something a month later – “Oh no, I didn’t read that…it’s gone.”*  NP01, non-participant, community support worker    *“Sometimes I get a bit mixed up with other programs. We get a lot of information through work, and I just read things through at the moment”*  NP02, non-participant, community support worker |
|  | **Facilitators of reach** | **Promotion and communication about program** | *“I have been thinking that if we knew a little bit more about the program, could we have sold it better? I just thought then because that’s the sort of program I think is really important is that encourages people to do something…I think that’s really valuable and I think it’s valuable for anything.”*  IS02, implementing staff, residential |
| **Adoption** | **Barriers to adoption** | **Cognitive factors** | *“If I can’t make a commitment to something, I can’t - I’m not going to do it because I won’t be able to put in 100%, but I didn’t want to waste your time doing that when I’m not going to do it, you know?”*  NP03, non-participant, community support worker  *“I’m really bad like that because it’s like – my time is my time and it’s like I don’t mix with people I work with, because I really don’t – I don’t see them. It’s not like we go to work and we see each other for eight hours a day. We’re very autonomous in our work life. So it’s like I’m used to not seeing them. And when I see them it’s like, “Hey, how are you going?” This is not to say they’re not nice people. They would be probably nice to hang out with, but I keep my work life separate from my private life, if that makes sense.”*  NP04, non-participant, community support worker |
|  |  | **Competing priorities** | *“I think people were interested, and I was at the meeting that that was presented at. People were interested enough to listen and actually hear what was going on. It's just the logistics of trying to get a timeframe that's suitable, and then an activity that fits a lot of people, to come together. To make that successful, you'd really need somebody who would pin down a time and say, "Okay, let's this, this and this one, join in and be an advocate and say they're doing it with you." But you'd need more than one particular time to be able to get people to join in. So, for instance, you might need a Tuesday afternoon, a Thursday morning and, I don't know, a Saturday afternoon or something, to try and fit around the fact that people's lives are so disrupted by shift work, and children, and partners working, and like, there's just so many variables. But you need a real advocate that's going, "Okay, I've got this, and I'm coming with you." Because, sometimes it can be a bit like sheep, one person says they're going and then a few others will join in.”*  IS03, implementing staff, residential  *“It’s stuff that prevents us from being involved. Yes, so I guess if it is stuff run at specific times during work hours, at specific places, it makes us really – it’s almost impossible. If it’s – yes, I guess – yes, a lot of the support workers in my position are reluctant to take any customer time off, and – because we feel like – well, obviously we’re not getting paid too, but we feel like we’re letting them down. Even if it’s something for ourselves, we’d much rather schedule it in our own time, after work or over the weekend, so it didn’t have to impact on customers.”*  NP01, non-participant, community support worker  *“And again, I don’t have time - I just wish I had time for me. The only time I have off, when the kids go to bed and I watch my shows, and then I have to go to sleep. So I just - I do try and focus on the weekends catching up with friends at night-time with them coming over, in between the sports during the day, et cetera. And that’s my ‘me time’ to say, ‘okay, I’m seeing the girls’, or, ‘the family’s coming over’, or we’re visiting somebody else or going to a birthday party. So yeah, that’s it.”*  NP03, non-participant, community-based worker |
|  |  | **Independent behaviour management (current activity levels)** | *“I play badminton, yeah and, at the moment I'm doing yoga as well, while there's still teachers here. We've had a few months where we've missed, but a new one's started again, so yeah, I'm doing yoga as well, yeah. And, I choose to walk or ride to work, and I choose to walk or ride to the shops, unless it's a really big load, so yes, I am reasonably active, and I am 62, so I'm not too bad.”*  IS03, implementing staff, residential  *“We’re on our feet all the time. I do anything up to 15,000 steps a day. I don’t feel like coming home and doing more physical work.”*  NP05, non-participant, residential care worker |
|  | **Facilitators of adoption** | **Nature of program** | *“That [a similar sort of service that was ongoing] would be good. That would be good because that way I would be able to say, ‘Okay, I don’t have time now, but in the future, near future, I’ll want to’ - yeah, so that’s what I want to do. If they’d had a gym or something that was local, that’s fine. That would be good.”*  NP03, non-participant, community support worker  *“I’ve never actually – I’ve worked in lots of different industries in my life and I’d probably say this is the first industry that I’ve worked in that offers like all sorts of different supports, networks, to their employees. So even if you –whether you choose to use them or to access them or not, that’s to your discretion. But at least we’re always made aware that they are there if we need them.”*  NP04, non-participant, community support worker  *“You know, I mean, a good idea may be to get these things out there would be maybe trying to organise things in advance, because we have team meetings and if things could be organised in advance to know - you know, like dates and what's happening and what have you, maybe then it could be addressed in the team meetings. Because they're compulsory, and so everybody would have to listen to whatever was going on, so then the awareness would be there, then. That's the only thing I can think of.”*  NP04, non-participant, residential support worker  *“And keep it – you could have your family ones, too, where – something – even though it is a bit more physical – like, we did lawn bowls, and stuff like that. But to me that wasn’t really physical anyway, that was just fun. So keep it fun, but – as in wellbeing for our brain too, we get enough workouts at work. Yes.”*  NP05, non-participant, residential care worker |
|  |  | **Nature of promotion** | *“I know…often put out information and we go, “that will mean nothing to them,” the way you worded that, that will mean nothing to them, you’ve got to, and it’s not dumbing it down, it’s just changing it to make it relevant. When people see that much information, they think, really, and people want it fairly quick and again, what’s in it for me?”*  IS01, implementing staff, community  *“But we just think yeah…not realising how much it could actually benefit us as individuals. Because, even I'm sort of thinking now, well I'm learning more talking to you now than I knew before, so yeah, I don't know how else you'd sort of bring things to people's fore. Unless you try and got somebody like [us] that could bring things, a little bit more information to staff and then be a bit more of an encourager.”*  IS03, implementing staff, residential  *“If you’re talking about physical activity, maybe don’t use it so much in physical activity – maybe just a walk, or something like that. Something that’s really down-paced, where we can just walk and talk, and – or take the dog for a walk, or something like that. Whereas we’re still doing physical activity, but we’re slowing it down. And when people think “physical activity”, too, they just think, “Oh, this is Hard Yakka gym stuff,” you know? So yes, to keep it a physical way, you’d have to try and slow the pace down. You know? Or, you know, keep it interesting. Okay, you go for a bush walk – we go for a bush walk or something like that.”*  NP05, non-participant, residential care worker |
|  |  | **Site/local level input, involvement and support** | *“We would’ve been a good resource for you because in a lot of cases, we’ve known these people for a long time and there’s that trust element. So, often, if I have to read through a lot of information myself or do something, but if someone who I really trust says to me, “this is a great thing, you should just try it,” you just go, okay, I’ll give that a try.”*  IS01, implementing staff, community  *“Come down and ask people, turn it the other way around. Rather than…creating something new and handing it down, start the other way, I always think it’s the wrong way around. You should be talking to people, obviously we need to look at the science of it and make sure it all works but I think people have to feel empowered about - they make suggestions about what they want to be involved in and then, I am sure they would be more likely to take it up, if they had a say in it. If they had some really, genuine input.”*  IS02, implementing staff, residential  *“And it’s just in the past few years with a lot of even the call centre girls going into the city to work, we used to have a team [in the area], and you knew the girls. And you could even go in and have a 10-minute chat with one of them. If you had a bit of a problem, they’d listen, you know, you’d go in and see so-and-so…or you could share good news and that, and there were lots of advisors that you knew, and now if you go into the office. I was there yesterday. Apart from the actual support worker team managers, there was only one advisor that I knew. And it’s just – you don’t know who to approach either for things. And well, I know some of those girls, they sometimes say, “Oh this weekend, we’re just going to have a get together and walk our dogs.” And you knew a little bit more that was going on; and it was easier to join in things like that.”*  NP02, non-participant, community support worker |
| **Context** | **Work culture** | **Engagement and morale** | *“I think it is engagement, I really do. I think people come, they do their job, they go home, unfortunately and socially we struggle sometimes to get a decent number of people at a social event. I don’t know what it is, there is a degree of cynicism around with the ‘us and them’ kind of feel, with the organisation and what people are doing.”*  IS02, implementing staff, residential |
|  |  | **Expectations of the organisation** | *“I think and if they want fitter and better employees…provide something where staff can actually have a say about what they would like and then actually perhaps put some money into it, making sure that happens.”*  IS02, implementing staff, residential  *“We go and it’s like we are the base of [the organisation] and what we do is amazing, each and every one of us, and I think what we do need to celebrate a bit more than what it is. And that adds to the health and wellbeing too, because if you’re made to feel special, it’s amazing how that works, like that will work for – that will bring a person’s esteem up and make them feel better. Because at the end of the day it’s all about feeling good and being good to you.”*  NP04, non-participant, community support worker |
|  |  | **Local or site level culture** | *“It’s how to get them in and I think when you go anywhere, it’s not the senior management, it’s actually the sergeant at arms, it’s the actual people because we go to the meetings and they will listen to what we say because mostly, they trust what we say.”*  IS01, implementing staff, community  *“What also is really good about our team leaders is our team leaders used to do our job, and previously the rostering staff used to do our job, and the advisors used to do our job. And anyone that used to do the job of the people that they’re now in charge of is so much better at their job.”*  NP01, non-participant, community support worker |
|  | **Work population – individual factors within the work context** | **Work stress and mental health within the population** | *“I think unfortunately this has come from a mental point of view, this has come on some mammoth change of the [organisation]…So, if anything, our staff are probably more hanging off the rafters and are more unsettled than they’ve ever been in their history. So, unfortunately from a mental point of view, probably any health benefits have been well dispersed, but I think that anyone would know that if you’re healthier and if you’ve lost a bit of weight, then you cope better with any change that life throws at you. So, I’m hoping that the people who did, who were part of this trial, whatever health benefits they’ve got have made whatever’s happening to them easier.”*  IS01, implementing staff, community  *“Some of the behaviours of our people, some of our violent dementias, and some of the mental health issues that the residents have got, that's probably a factor in staff wellbeing too.”*  IS03, implementing staff, residential  *“And I just hope for the best that things do change with the [restructure] and there’s more work when everything goes live because it’s just hard. People are struggling. People are really, really struggling with all the changes.”*  NP03, non-participant, community support worker  *“Because there’s a lot of changes I’ve decided I’m [going to cut down the work I’m doing] because you’ve got a whole new range of people that we’re working with, and for me to mentally, emotionally and physically cope with all of that change.”*  P13, program participant, community support worker |
|  |  | **Personality, innate caring** | *“With a lot of the people we’ve got, they put their families first, their customers first, their pets first, their everything and they come at the end of the line and they do so for a variety of reasons and that’s probably why they’re good at their craft and they’re drawn to aged care is because they’ve got that type of selfless personality and although they’re quite an eclectic group of people, they do have that same personality trait that sort of runs over and over different facets in different ways.”*  IS01, implementing staff, community  *“A lot of people in a particular job, like a caring role, also are in caring roles outside of work, because they fit that particular personality type. So a lot of our staff are caring for their parents, grandchildren, or children themselves. And a majority of our staff are also women. But I think, overall, our staff will put everyone else and everything else first before themselves. So – and I don’t know if it’s a female mentality, or just a carer’s mentality, but we’ll always put work and family and – so doing something for themselves is probably the last thing on their mind, honestly.”*  NP01, non-participant, community support worker  *“Because this work that we do, it’s very – it’s all-consuming. It’s mentally, physically consuming. Emotionally it’s very consuming and you know what? It is tiring. It is tiring what we do. It’s not just about turning up. We talk to people. We listen to people. We do stuff for people. and it’s very it’s exhausting, it is, when you – and I think it might just be a nicer outlet if the support workers had that outlet where they could go and learn to – well not learn but sort of get some assistance and maybe getting some time back for themselves.”*  NP04, non-participant, community support worker |
|  | **Work structure** | **Changes in the work environment, culture, structure** | *“We used to have a really full office full of people, and we never actually had designated team leaders, but we sort of had a supervisor, but she kind of looked after the rostering team, and the rostering team kind of looked after us. So we were – and because we had to jump off timesheets. That’s the other change, too. We physically had to visit the office every week, and so we got to know all these people, rather than just a name on the end of the phone, and we saw these people all the time. So there used to be a social connectedness with people during their work hours, which made more people likely to attend outside of work hours. So as much as some of these things sound really interesting to people, you’re not as drawn as what you used to be, because you have no social connectedness as much anymore.”*  NP01, non-participant, community support worker  *“We all used to work out of this office…and it was a lovely time because you'd always be popping in there to get out your rosters and things and you'd run into people. You'd run into the office staff and it was a really lovely atmosphere and that's all gone now.”*  P10, program participant, community support worker |
|  |  | **Relationship with or isolation from work peers (and logistics of getting everyone together)** | *“When I was younger, I wanted to be a bit more involved. And one of my biggest drawbacks was, I’d say to other support workers, “Oh, let’s get together. Not talk about work. Just have a night out or do something.” And the two or three that did get back, one would say, “Oh, let’s make it in the morning.” And another one would say, “Let’s make it in the afternoon.” And another one would say, the evening. And you just couldn’t get a time for everyone to get together. I have found as a support worker, it’s quite a lonely job. You do get out and meet your customers, but you don’t have your peers to talk to in the same way as you work in an office or factory or a shop or something.”*  NP02, non-participant, community support worker  *“I’m really bad like that because it’s like – my time is my time and it’s like I don’t mix with people I work with, because I really don’t – I don’t see them. It’s not like we go to work and we see each other for eight hours a day. We’re very autonomous in our work life. So it’s like I’m used to not seeing them.”*  NP04, non-participant, community support worker |
|  |  | **Physical work and impact on health** | *“We work all day, and it’s a physically demanding day. We just don’t want to do anymore physically demanding things…but there’s some days that we just come home exhausted from work, and to actually think about doing more physical work – it would be a drain to even think about it.”*  NP05, non-participant, residential care worker  *“I mean, the job itself is exhausting. So, yeah, I do. I mean, a few people who I work with, they say when a shift is over, "we're just exhausted." So, I don't think…if people did see it [the program], they're probably like, yeah, no thanks. You know, you just want to go home from work and just sit back and relax. You don't want to be doing any extra. From just talking with my work colleagues, that's the picture that I get.”*  NP06, non-participant, residential care worker |

1. Feasibility (adherence, subjective outcomes, program components, and sustainability) of the Activity for Wellbeing Program in frontline aged care workers

| **Overarching theme** | **Theme** | **Sub-theme** | **Selected quote (s)** |
| --- | --- | --- | --- |
| **Adherence** | **Barriers to adherence** | **Competing priorities** | *“I felt really, really super excited, as I’m sure that you’ve written in there, at the start; I thought ‘I’m going to be a success’ and whatever. But as usual, life gets in the way for a number of reasons and your work - if I had a nine to five, normal job life might be a bit simpler, but because my work hours change pretty much on a daily basis, it’s often hard. I made a change at the start where I blanked out some time for work, which was awesome. Then that obviously got changed down the track where I needed money and there was work offered at that time, so it’s then trying to make the effort to - on your day off, ‘I’m going to go to the gym on my day off’, or I’m needing to make time to go on the weekend, which I’m still working on. I haven’t actually got there yet.”*  P25, program participant, community support worker  *“Everyone’s got different schedules. We’re all different ages and we’re all different levels of life let’s say. Some of them with young children. Myself I’ve got older children, I’ve got a grandson that I have just about every weekend and I go to church on a Sunday so it leaves me with a Saturday to try and get everything done that I don’t do during the week.”*  P07, program participant, community support worker |
|  |  | **Environmental and Accessibility issues (natural or exercise environments)** | *“I think getting out and actually doing things, and I really like to get out outside and do things, it’s the weather that is really cold and wet. I’ve been back on the cross trainer which is good but I feel like on the cross trainer the only thing I’m doing is getting exercise, I’m not benefiting in any other way if you understand what I mean?”*  P06, program participant, residential care worker  *“I'd say the last two weeks, the weather, and I'm a bit surprised because I'm one of those people who always say, "I love winter." But when I look at my steps, I realise I don't really do much activity at all, whereas in the warmer weather, I was out and about a lot more, which surprised me. But often, just with my clients and work, often I'll just park up their driveway. A lot of the time now if it's not raining, I'll park down the street and get in a few hundred steps. They're the days I'm really conscious of trying to build up my steps. If I didn't have my pedometer on, I wouldn't even bother.”*  P10, program participant, community support worker  *“Yeah and I did ask about that [the gym] but unless it’s during working hours, and now they’ve been promoting more they’re getting, the gyms it’s quite full…And I mean they opened up the great big new gym complex down, that’s [in one area], which isn’t feasible for distance wise because I live [in another]. But once again for us to use the equipment after hours, that’s not, it’s a no go.”*  P20, program participant, community support worker |
|  |  | **Individual factors** | *“Yeah, I think it does. Some people are straight away but I think - and others - like, I know me. It takes me a long time to talk myself into it, get my head around everything. I think stop making excuses and just get in there and do it. Then I think if I'd started back [when] we met?”*  P04, program participant, community support worker  *“Yeah, I’ve been doing it and thought “Oh, I’ve heard my own voice. Yes! I can do it.” It’s like if I’ve got barriers there, you’ve got somebody else there and it’s almost as if “Oh my goodness, I am making excuses for myself.” If it’s in your head you just fight with yourself but having somebody else there and like I say bouncing back off it’s like “Yeah, I’m making excuses.”*  P07, program participant, community support worker  *“From my side, I'm thinking I could be a lot more active, but I’m not. I should motivate myself more, that type of thing. But I don't and I'm a knitter and I sit there and knit a lot. Sometimes I think, 'I should be out there walking around doing a bit,' but I don't.”*  P10, program participant, community support worker  *“Because I’d gotten used to the [first gym], and then I thought ‘oh, this [second gym]’, there’s no-one there; can’t get anyone to help you. And I think I’d joined up on the ‘I’m going to do it’ because I didn’t want to lose that continuity of I’m going to stop again. And the whole thought, went in there and gone ‘oh, how am I going to do all this again, is it going to be the same equipment?’ or whatever, and I’d sort of temporarily lost the mojo for a while of going ‘yeah, I don’t know’. And so then you sort of offered to do that and I’m like ‘oh’…‘am I asking too much for that to happen?’”*  P25, program participant, community support worker |
|  | **Facilitators of adherence** | **Having accessible and comfortable environments** | *“And I like to go for walks, to take the dog for a walk because we live in a rural area. That is really enjoyable to go for a walk with him. And I try to, because he needs the exercise, I need the exercise but it’s not something that I will do or will have time to do every day. So I’m trying to get into that habit of building into my day exercise, because I can feel the benefit of it.”*  P06, program participant, residential care worker    *“You feel welcome [at the gym] and they’re always happy to see you and always say goodbye and that sort of thing. They’re always around talking about what you’re doing and that sort of thing, and even with a lot of the other participants in the gym too, they actually talk to you where a lot of them don’t up at the other one. I enjoy travelling from home up there. I’m going three times a week now.”*  P09, program participant, volunteer community worker  *“I don’t know about other people but having you available at any time to be able to ring…that was the most helpful, and having the pedometer, which made me aware of working that little bit harder to get my steps in. And knowing that the availability of going to a gym and everything is there was just really me taking those first steps to do it. But I was still quite happy with what I was doing at home and I was just working around my health issues and still am.”*  P15, program participant, community support worker |
|  |  | **Overcoming barriers to activity** | *“I'm not going to be doing that [joining a gym] until later in the year, only because I'm just not going to be able to afford gym membership until then, but my eating and I've been - you know, I try to exercise - like, do something every day now.”*  P04, program participant, community support worker  *“Because I have so little time, what I am doing now is when I am working, I’m more aware of doing things that are actually exercises in my work. Instead of bending over, doing squats and then stretching my legs and parking further away from the shop and just walking. And instead of driving to every shop I need to go to, I park in a central location and walk everywhere. So instead of parking close, I get more walking.”*  P06, program participant, residential care worker  *“Well on the first meeting we spoke about having goals or things that I would like to do. I did mention that I wanted to do maybe aqua aerobics. But I did go to one but I found being indoors the way it was with the chlorine and the heat it was a bit overwhelming…so I discarded that. My walking which I have programed to do, started off maybe about a week but then my friend I’d walk with she did an ankle injury so that sort of put a stop, and I didn’t motivate myself to do it on my own, and weather and that. But I was very happy with the membership, when I was going down to the [university] gym.*  P20, program participant, community support worker |
|  |  | **Personal motives** | *“I want to get myself fitter and healthier and, like I said, going through summer and this is the biggest I've been since I was pregnant and I felt really uncomfortable. Yeah, everything's getting hard. Even at work I found that I don't - I'll be sort of half cleaning and half shopping and social and stuff like that, appointments and whatever else and personal care, but, when I was doing the cleaning, I even found bending down to do the showers properly, I'm like, "Oh, this is getting hard." Yeah. Getting back up again, my legs were like - and I've always had really strong legs but I think they're carrying too much now. I have to change. I have to. Yeah. So I think I'm getting myself - you know, especially in the last few weeks I'm starting to feel I can do this.”*  P04, program participant, community support worker  *“Just thinking about when you came to the office and you did that test, like walking back and forth to gauge my fitness level. That was helpful to me. And I also am more aware of health issues related to exercise. If I see something, I know I should be exercising and getting this - this is happening because I’m not exercising or if I’m inconsistent with my exercise, you know what I mean?...It inspired me, I guess you could say, to do better.”*  P06, program participant, residential care worker  *“I think also working with the elderly here I see some of them how through the years they can’t do anything and it’s like I can still do it so do it while I can.”*  P04, program participant, community support worker |
|  |  | **Social factors and support** | *“Actually, yeah, what I'm going to do with them [the kids] this week is there's actually some dance exercise things on YouTube, so I'm going to put a couple of them on because you know - and technology the way you can put everything just on the TV now, I'm going to put some of them on the TV and I'll get them to do them with me during the week, because they love dancing around, so that'll be a bit of fun.”*  P04, program participant, community support worker  *“Maybe if I had someone to do it [the gym] with too. That sometimes is better, like a buddy. I was a bit like that when we were doing our exercises through the other gym. I didn’t really but now I have still got five of those left and [partner] said, “Well, maybe we could just go at a different time.” You know, when he is not so busy but it is just finding the time to do it now because of what I am doing at home.”*  P15, program participant, community support worker  *“Well if you’ve got a group, well obviously in our line of work it’s very hard to get everyone that works at the same company at the same time, that was the time issue I suppose for everybody. But to me if you’ve got someone or a group you tend to push yourself a bit better, or make you more motivated because you are doing it with other people.”*  P20, program participant, community support worker  *“So by doing that, rather than just joining the gym and going ‘yeah, I’ll get around to doing it’ and wasting my money, so yes, obviously somehow must have made a phone call to you, and then you’ve probably called me for an appointment to sign up, and I felt that was probably a good thing because then I had to commit to it. No-one held a gun to my head, but it was a good thought that ‘yes, I want to change my habits and yeah, let’s do this’.”*  P25, program participant, community support worker  *“Well, actually a few of the school mums are on that [web page], so I just joined that. I'm not joining their challenges and stuff like that or doing their shakes or anything, but just a lot of that comes - a lot of people - there's thousands in this group and just reading that it's like well, so many people are in the same boat. So many. And then there's other people that just - they've got so many more issues than I've got, like as with family. Kids with special needs and they work full - you know, I'm reading all this stuff and I'm going you know, I actually haven't got it that bad. And if they're doing it, why aren't I doing it? That sort of thing. So I've really found that interesting.”*  P04, program participant, community support worker |
|  |  | **Taking charge, independence with behaviour** | *“I might have adapted something differently at different times. When I’ve stopped going to [the gym], so I’ve tried to sort of compensate and do things.”*  P20, program participant, community support worker  *“So by doing that, rather than just joining the gym and going ‘yeah, I’ll get around to doing it’ and wasting my money, so yes, obviously somehow must have made a phone call to you, and then you’ve probably called me for an appointment to sign up, and I felt that was probably a good thing because then I had to commit to it. No-one held a gun to my head, but it was a good thought that ‘yes, I want to change my habits and yeah, let’s do this’.”*  P25, program participant, community support worker  *“I know you’ve done the newsletters and information and things like that, but quite honestly, I think the general public know that there is a lot of information about fitness, and there’s a lot of places you can go and do things. It’s just making that hill climb of the decision to get it happening. That’s it. How big a priority is it in your life, are you going to commit and put some time into it? Get your priority right and you can make it happen.”*  P25, program participant, community support worker |
| **Subjective outcomes** | **N/A** | **Mental health** | *“But what happened was doing this program, is this program actually…helped me reach my – it was my mental senses, you know my state of mental health and my sense of – it motivated me. So it motivated me to be more active about everything and one of the problems that I’ve had with this depression and anxiety thing, is pursuing the things that I love”*  P01, program participant, community support worker  *“I can see the benefits of what it’s done for me improving my mental health and physical health. I sleep better and I’m enjoying life a lot better. It’s definitely a plus. I can see the relationship between the exercise and mental health; helping to improve it.”*  P09, program participant, volunteer community worker |
|  |  | **Motivation and mindset** | *“Well I'm setting myself goals. I'm doing that and it's just got me thinking. It's always in the back of my head now. Always in there and yeah, it's hard to get out what I'm trying to say. Definitely it has; it's given me the motivation, and I have started thinking outside the square too and it's got me thinking about more ways that I can try and do things and make it fun.”*  P04, program participant, community support worker  *“It got me motivated, I know that much, to get out. Got my Fitbit on and if I don’t get to that 10,000 for the day then I feel quite deflated so it’s go home, do some exercises, use my little hand weights, use my balls so it has motivated me.”*  P07, program participant, community support worker  *“It [the program] was an opportunity to be spurred on to actually doing something about it, instead of just mentally assenting to it. To get over that hurdle of actually getting into some more physical activity is the biggest issue. So for me, I think it was that spur that I needed to sort of get started.”*  P13, program participant, community support worker |
|  |  | **Physical health** | *“Yeah, I think about getting outside amongst the trees and the birds and the actual exercise, the actual walk, yeah I feel good. And the exercise, after I was coming out of that hole where I wasn’t exercising, it was a bit more strenuous. But now it’s better, much better. I would say good. I felt really good about it, good in terms of physical exercise and not being out of breath.”*  P06, program participant, residential care worker  *“Look, it’s improved my life and I feel better for it. When I look back, I’m like, ‘God, I got myself in that state,’ and now it’s, ‘Let’s get on with life.’ I actually feel like – well, I’ve definitely been given a second chance through this program and also no sort of ending up in hospital.”*  P13, program participant, volunteer community worker |
|  |  | **Positive emotional response to program**  (i.e. encouragement, pride, feeling valued) | *“It encouraged me to do exercise because I’m so busy and I have so little time to myself, that exercise is something that gets put as a lower priority on the list. So it encouraged me to get out there and exercise and think more about what I’m doing in exercising.”*  P06, program participant, residential care worker  *“But I guess it’s just mainly the satisfaction of knowing that I have done this myself – I mean with your help – and I am going to stick to it. Yeah. I am going to stick to it, and there will be times that I will probably lapse, and there’s probably times that I will do more but I know that it’s all there and it’s available to me.”*  P15, program participant, community support worker  *“They’ve gone [the things I set out to achieve], blown it right out of the water. The upper body strength is great, and there’s no way in the world I could have been pushing – on some of the machines, I’m up to 70 kilos at this stage, and the same with leg strength. On the horizontal press I’m doing 100 kilos, and it’s just blown me away, and the same on the squat machine; what I’m doing on there now amazes me. I’ve definitely got out what we started out to achieve.”*  P09, program participant, volunteer community worker |
|  |  | **Positive perceptions around physical activity and health** | *“I think I generally do quite well but every now and then I fall off the wagon, as I have with my eating. That's the thing that starts it and I've just come to terms with it, "Well, we all do that. It's not the end of the world." I've learnt not to be so hard on myself. I think that's it. I get very hard on myself.”*  P10, program participant, community support worker  *“And I think basically it made me more aware of nutrition and choosing the right foods, is the main thing. Because everyone sort of things it’s a quick fix of dieting or something, but no I just sort of had made myself more aware of what foods I actually am eating, you know without going without so to speak. It’s just broadened my aspect I think. Deep down you know what things to do as far as exercise and nutrition et cetera it’s just the motivation.”*  P20, program participant, community support worker |
| **Sustainability** | **N/A** | **Having something in the organisation on a broader scale** | *“That [a similar sort of service that was ongoing] would be good. That would be good because that way I would be able to say, ‘Okay, I don’t have time now, but in the future, near future, I’ll want to’ - yeah, so that’s what I want to do. If they’d had a gym or something that was local, that’s fine. That would be good.”*  NP03, non-participant, community support worker  *“I was hoping that it would continue. We felt like we had somebody like on our side let’s say that looks at us not just as employees. For our own health, our own safety, just a bit more personalised not like an employee, a number. You know what I mean?”*  P07, program participant, community support worker |
|  |  | **Other workplace initiatives** | *“Being more aware of what to do and just counting your steps and all that sort of stuff. [We were given] little pedometers years and years ago and we did a healthy well-being thing but this was back like eight or 10 years ago. And yeah. No, that was great but there was no follow up if you know what I mean. But it is still making you aware to keep you fit when you are working. Like if you were cleaning or you were taking people out. Learning how to get people in and out of cars and yourself as well, and taking things like wheelchairs in and out of your boot and all that. Yeah, being aware of looking after your own body as well as being mindful of somebody else’s.”*  P15, program participant, community support worker |
| **Program components** | **N/A** | **Need support and affect** (intensity regulation, affect, no pressure or judgement from AEP) | *“It triggered things. It triggered more thought. It triggered more subtle thought. To really dig down. So, “How do I really feel about this?”, “Why do I feel like that?” How come it’s really easy for me to feel good when I’m doing exercise; and I can monitor myself really well, I’ve never harmed myself during exercise and I always feel good, and I know when I feel bad, I get over it very quickly because I actually know that this is building muscle, quite literally, and it’s also building my psychological resilience, which is something that a lot of people don’t understand.”*  P01, program participant, community support worker  *“For me it's been perfect because I've always felt if I need to ask something or I've got a problem, I could contact you and yeah, that's just the way - I would have no hesitation and there have been times I've contacted you and I haven't given it a second thought. I've just contacted you and you've always come back and yeah, I'm very happy.”*  P10, program participant, community support worker  *“Well, the program has helped me tremendously and having you helped because I know I can ring you up at any time. That’s been great. And yeah, just the whole aspect of it. I feel as if someone is prepared to listen to me and cares.”*  P15, program participant, community support worker  *“You haven’t judged us, whether we’ve done it or not, you haven’t said ‘oh, you’re a rotten person, you should be up to doing five days a week by now, at least 5km every time you go out’. You haven’t done that to us, and I think that’s important as well.”*  P13, program participant, community support worker |
|  |  | **Self-management tools**  (action and coping planning, goal setting, self-monitoring and wearable devices) | *“Well I'm setting myself goals. I'm doing that and it's just got me thinking. It's always in the back of my head now. Always in there and yeah, it's hard to get out what I'm trying to say. Definitely it has; it's given me the motivation, and I have started thinking outside the square too and it's got me thinking about more ways that I can try and do things and make it fun.”*  P04, program participant, community support worker  *“I used to log in my steps every Sunday night [to the website] and I used to - I'd find that helpful. I could see the graph and see where I was going with that. That was good and just the texts I received from you. It was just a reminder of how - just a reminder of encouragement to keep going.”*  P04, program participant, community support worker    *“Yeah, that’s where I started off using those goals, and I’ve moved on a fair bit now since then. I’ve got a whiteboard with goals and settings on it, a small one next to my desk because I write what I want to try and achieve through the week. It was useful having the goals that actually helped with encouragement to keep going, or to get going, actually.”*  P09, program participant, voluntary community worker |
|  |  | **Exercise programming (home or gym exercise) and activity plans** | *“Yeah, a lot of structure to my exercise routine, like the gym and that sort of thing. Going in there and being shown machines to use for what I was trying to achieve and that sort of thing. So yeah, it’s given a lot of structure to that, and encouragement to continue and go for it.”*  P09, program participant, voluntary community worker  *“I guess, and the best part was getting you to come out to the gym with me and reacquaint me with the gym equipment that I’d probably forgotten. So that was excellent that you did that. I’ve got some sort of clue but there’s a lot of equipment changes that have happened in that sort of time, and my knowledge has obviously gone by the wayside somewhat, so by you showing me the exercises and writing that down to have that to go by because retention, as you get older, gets a little bit difficult.”*  P25, program participant, community support worker |
|  |  | **Newsletters, website and outcome measures** | *I've never been back on it [the website]…Only because I've got all that on my phone anyway. I've got like my S Health on there and that gives me summaries and it compares me with other people. Not names, but it will have a group for my age and whatever and what they're - you know, like it's got - it's very detailed anyway and I always make sure I do my 10,000 at least a day steps and 60 active minutes a day and things like that, so I was already sort of doing that anyway.*  P04, program participant, community support worker  *“Just thinking about when you came to the office and you did that test, like walking back and forth to gauge my fitness level, that was helpful to me…It inspired me, I guess you could say, to do better.”*  P06, program participant, residential care worker  *“Everything was like the tools were there and then you send through those email, you know, the newsletters, the little bits of information that I can get from that, that helped because I liked them coming through. I’m finally trying to get around to reading them. It might be two weeks later and I’ll have say 15 minutes and I’m sat in the car and it’s like ‘Let’s scroll through. Ooh, I haven’t read that yet.’ off I go. I never used to be able to get to them straight away but I leave them there and I scroll back and go ‘Right, yeah, okay.’”*  P07, program participant, community support worker  *“After I signed up and then I thought, 'Heavens, I have to get on the net or whatever,' because I'm not that tech savvy. But for me, I found it really quite easy. I have a tablet and I sit in there of a Sunday night and just pop them in. Each day, I write my numbers down in my diary and then of a Sunday night, I just put them in. And there's plenty of information there and it's so easy to find.”*  P10, program participant, community support worker  *“What didn’t work? Probably having to remember to take that watch off, and then you’ve got to have the chart next to it for what time you took the thing off, otherwise you’d leave it downstairs, you’d start drying your hair and go ‘oh my God, I’ve got to go and - was it 15 minutes that I had that thing off?’ You’d get better at it.’”*  P25, program participant, community support worker |
