# Supplementary material for: Feasibility and Process Evaluation of a Need-Supportive Physical Activity Program in Aged Care Workers: The Activity for Well-Being Project
Source: Front Psychol. 2020 Sep 30;11:518413. doi: 10.3389/fpsyg.2020.518413 (PMC7554301; doi:10.3389/fpsyg.2020.518413)
Supplement: Supplementary file 3 [file Table_3.DOCX]

**Supplementary Material 3** Change and Performance objectives as evaluated by the 3-month questionnaire: participant rated scores for the achievement of the change and performance objectives that were developed during the Intervention Mapping framework as scored on a five-point Likert scale (1 = ‘not at all’ and 5 = ‘very much so’)

| **Performance Objectives** | **Evaluated by:** | **Mean** | **SD** | **α** |
| --- | --- | --- | --- | --- |
| **Find time to undertake physical activity** | Do you generally feel you are better able to find time to be active? | 3.73 | 1.24 | - |
| **Find motivation to undertake physical activity** | Do you generally feel you are more motivated to be active? | 3.95 | 1.29 | - |
| **Identify opportunities to undertake physical activity** | Do you generally feel you are better able to identify opportunities to be active? | 4.05 | 1.13 | - |
| **Change Objectives** | **Evaluated by:** | **Mean** | **SD** |  |
| **Autonomy cluster** | - Do you feel that you are now better able to identify activities that are flexible to fit in with your schedule? - Do you feel that you are now more likely to view physical work as an opportunity to be active? - Do you generally feel that you are in control of your own activity choices? | 4.00  4.14  4.33 | 1.07  1.04  0.86 | -  -  - |
|  | **Composite Total** | **4.16** | **0.88** | **0.87** |
| **Competence cluster** | - Do you feel that you are now better able to build activity into your day? - Do you feel that you are now more confident in your ability to find time to be physically active, even on busy days? - Do you feel that you are now more confident in your ability to set activity goals? | 3.86  3.59  3.81 | 1.21  1.26  1.44 | -  -  - |
|  | **Composite Total** | **3.75** | **1.24** | **0.94** |
| **Relatedness cluster** | - Do you feel that you are now more likely to utilise opportunities to be active while with friends, family or work peers? - Do you generally feel that you have the support to be active? | 3.32  4.00 | 1.52  1.10 | -  - |
|  | **Composite Total** | **3.64** | **1.25** | **0.84** |
| **Excluded variable**  **(Positive Exercise Affect)** | - Do you feel that you are now more confident in your ability to control activity intensity? | 3.90 | 1.26 | - |
|  | **Composite Total** | - | - | - |
